# Synthesis of Chitosan Nanoparticles via Microfluidic Approach: The Role of Temperature in Tailoring Aggregation for Enhanced Uniformity

**DOI:** 10.3390/mi16060642

**Published:** 2025-05-28

**Authors:** Muqarrab Ahmed, Yangcheng Lu

**Affiliations:** State Key Laboratory of Chemical Engineering and Low-Carbon Technology, Department of Chemical Engineering, Tsinghua University, Beijing 100084, China; muqarrab54@gmail.com

**Keywords:** microfluidic device, flow synthesis, chitosan nanoparticles, aggregation

## Abstract

This study presents the synthesis of chitosan nanoparticles (CSNPs) using a microfluidic device. Microfluidic rapid mixing enables fast nucleation for small-sized nuclei, but a high PDI value like 0.956 shows uncontrollable growth of small nuclei, resulting in the formation of larger and more variable aggregates at room temperature. High temperatures play a key role in controlling the growth of CSNPs to enhance uniformity. Temperatures of 40 °C and 50 °C promote controlled interactions among small nuclei, while increasing the temperature to 80 °C further accelerated the curing process, suitable for synthesizing CSNPs with various sizes. At 80 °C, size regulation can be achieved by changing the TPP concentration, which controls surface curing and affects the size as well. These results emphasize the impact of elevated temperature and precise TPP concentration for product quality control and modulation in CSNPs’ synthesis.

## 1. Introduction

**Figure 1:**
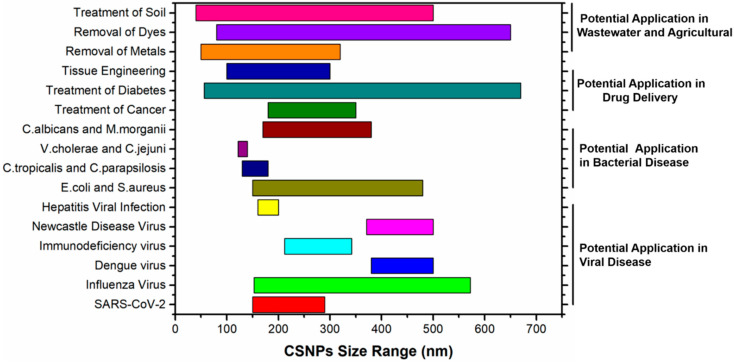
The size distributions of CSNPs for different potential applications reported in the literature.

Chitosan is a polymer known for its strong bioactivity, biodegradability, and non-toxic properties [1,2,3,4,5,6,7]. It is a polysaccharide containing reactive groups such as hydroxyl (–OH)- and amino (–NH_2_)-based groups [8,9,10,11,12]. Chitosan nanoparticles (CSNPs), in various sizes, are increasingly in demand for a wide range of applications across multiple fields. In wastewater treatment, they are used for metals [13] and dyes removal [14] and pathogen control [15], offering an effective solution for environmental cleanup. In agriculture, these nanoparticles are applied in soil and seed treatments to enhance plant growth and protect against diseases [16,17,18]. In food preservation, they are incorporated into packaging materials [19,20], helping to extend shelf life and maintain food quality. CSNPs are also used in skincare products for their ability to improve skin hydration and deliver active ingredients [21,22,23]. In the biomedical field, CSNPs are used in biosensors [24,25], bio-imaging [26], viral diseases treatment [27,28,29], cancer treatment [30,31], diabetes treatment [32], tissue engineering [33], wound dressing [34,35], and antibiotic drug delivery [36], etc. The tunability of CSNPs’ size is critical for application-specific performance. For instance, drug delivery applications require sizes of 180–200 nm (cancer therapy) and 180–250 nm (diabetes), while viral applications require 150–200 nm (COVID-19), 180–360 nm (Influenza), 370–500 nm (Newcastle virus), and 380–500 nm (Dengue). The size distributions of CSNPs reported in the literature are summarized in Figure 1, classified with different potential applications. The corresponding studies and their brief information can be found in Tables S1–S5. As is well known, size is of great importance for the application of nanomaterials. Such a wide size range in the literature reflects an insufficient ability to control the size.

Widely employed for CSNPs’ synthesis, the ionic gelation method relies on electrostatic interactions between the positively charged amino groups of chitosan (CS) and negatively charged sodium tripolyphosphate (TPP) ions [37,38,39,40,41,42]. While offering operational simplicity, this method remains challenged by poor control over product size and morphology. Specifically, traditional batch processes suffer from inefficient mixing and reaction homogeneity, leading to substantial batch-to-batch variations and scalability issues [43,44,45]. Additionally, the gradual addition of crosslinker to the polymer solution can create localized high-concentration regions in later stages, disrupting interactions among preformed nanoparticles [46,47,48,49]. Previous studies addressing CSNPs’ uniformity have primarily focused on post-synthesis treatments like filtration or centrifugation rather than optimizing the synthesis methodology itself [50,51,52,53,54,55,56]. As an alternative, recent efforts have explored enhanced mixing strategies and continuous preparation techniques. For example, Majedi et al. utilized a flow-focusing microfluidic device to prepare CSNPs for fuel-cell applications, examining the effects of variable flow rates on mixing efficiency [57]. Greco et al. employed an S-shaped microfluidic platform to synthesize CSNPs for macromolecules delivery [58], while Siavashy et al. developed chitosan-coated magnetic nanoparticles via microfluidics for cisplatin drug delivery [59]. Microfluidic devices with submillimeter-scale channels (internal diameter ≤ 1 mm) [60] enable rapid, controlled mixing in continuous flow, minimizing secondary interactions between excess reactants and pre-formed particles. This approach has demonstrated promise for efficient, reproducible nanoparticle synthesis across diverse systems, with strong potential for scalability [44,45,61,62,63,64,65,66]. A suitable microfluidic channel size and flow rate are effective parameters for optimizing mixing. As noted by Svenja Schmid et al., the use of a 0.5 mm T-junction with a flow rate of 10 mL/min is an effective configuration for achieving enhanced mixing efficiency between reagents in microfluidic systems [67]. However, the ionic gelation approach for CSNPs’ synthesis still lacks a comprehensive understanding of aggregation mechanisms and effective strategies to mitigate particle agglomeration, limiting their translational potential in practical applications.

In this work, we employed a microfluidic-based ionic gelation process for the preparation of CSNPs, with a focused investigation into how temperature and cross-linker concentration influence product aggregation. Our study aimed to elucidate the underlying aggregation mechanisms and systematically optimize synthesis conditions to achieve maximal uniformity, thereby enhancing their suitability for various potential applications.

## 2. Experimental Methods

### 2.1. Materials

Chitosan (CS, MW = 100 kDa), sodium acetate anhydrous (99.90%), and sodium tri-polyphosphate (TPP) were purchased from Meryer company (Shanghai, China). Acetic acid (>99.5%) was purchased from Tokyo Chemical Industry (Tokyo, Japan). All the chemicals were used without further treatment.

### 2.2. Characterization Methods

Transmission electron microscopy (TEM, JEM 2100 F, JEOL, Tokyo, Japan) was used to observe the morphology of the CSNPs. Dynamic light scattering (DLS) was employed to assess the average size and polydispersity index (PDI) of the CSNPs.

### 2.3. Equipment and Procedures

The CSNPs were prepared with the ionic gelation method by the electrostatic interaction between the chitosan polymer and sodium tri-polyphosphate. When using a buffer solution of pH = 5 prepared with acetic acid (0.158 wt%) and sodium acetate (0.443 wt%) to dissolve chitosan and TPP separately, the amino groups in the CS can be pronated to obtain positively charged CS, and the TPP can be dissociated into negative phosphoric ions (P_3_O_10_^−5^). Mixing the two solutions will start up the gelation process due to the static interaction under a pH controlled at 5.

**Figure 2:**
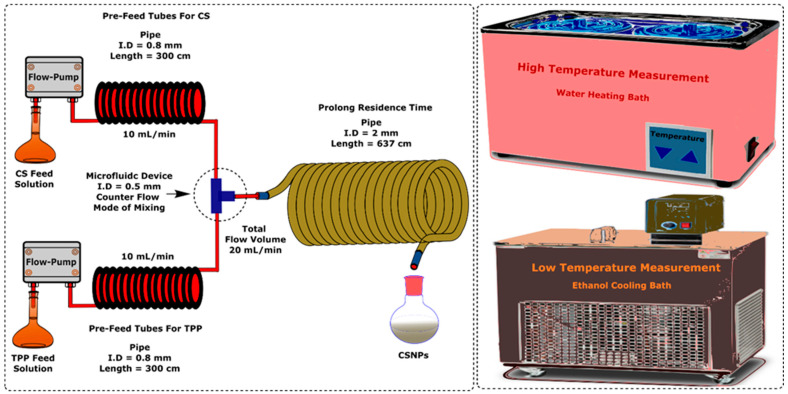
Schematic diagram of a continuous synthesis system for the fabrication of CSNPs.

Figure 2 shows a scheme of a continuous synthesis platform for CSNPs. It includes a T-junction with a 0.5 mm internal diameter and a delay tube with a 2 mm diameter and 637 cm length. This configuration was chosen to promote rapid nucleation from the initial mixing and ensure stable flow without considerable back-mixing throughout the downstream. To control the temperature, the feed bottles, 300 cm pre-cooling/heating tubes, the T-junction, and the delay tube were immersed in a thermostat. More information about the platform and temperature control evaluation can be found in Figures S1–S5. Two constant flux pumps (Xingda, Beijing, China) were used to deliver the CS and TPP solutions with a flow rate of 10 mL/min separately. The platform worked under ambient pressure. The temperature and feed concentrations of CS and TPP were adjusted on demand. At least three experiments were repeated to ensure the reliability of the experimental results.

## 3. Results and Discussion

### 3.1. Tailoring the Fabrication of CSNPs: Insights from Temperature Variation

#### 3.1.1. Room Temperature

**Figure 3:**
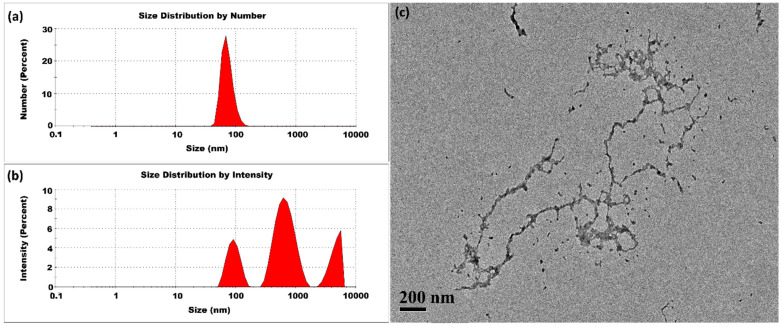
CSNPs synthesized at room temperature with a CS:TPP ratio of 5:1. The final suspension was analyzed directly. (**a**) Size distribution by number percentage; (**b**) size distribution by intensity percentage. (**c**) TEM diagram.

The experiment was first conducted at room temperature. The synthesis of CSNPs was performed with a CS-to-TPP weight ratio of 5:1, corresponding to a CS concentration of 2 mg/mL and a TPP concentration of 0.4 mg/mL. Figure 3 presents typical DLS and TEM measurements. The size distribution by intensity percentage indicated the presence of small-sized CSNPs, along with two additional peaks representing larger aggregates. Herein, we used Z-average method to measure the average size and polydispersity index. Because the scattered light intensity is proportional to the square of the particle volume, the Z-average size is significantly skewed toward larger particles. The CSNPs prepared at room temperature had an average size of 368 ± 10 nm and a high PDI value of 0.956 ± 0.024. Post-treatment of CSNPs also revealed the presence of aggregates with variable sizes, as seen in Figure S7. These findings implied that to stabilize the small nuclei and prevent aggregation, a rapid surface curing process might be essential for limiting their aggregation. The presence of small-sized CSNPs alongside aggregates indicated that some nuclei achieved complete curing, while others remained uncured. This uncured fraction contributes to a diverse range of sizes. In addition, a TPP concentration of 0.4 mg/mL was identified to be enough for the complete conversion of the CS feed of 2 mg/mL, as evidenced by the carbon equilibrium between the fed CS and the CSNPs separated out from the product suspension. Herein, the ratio of CS to TPP was not higher than 5:1 to guarantee the sufficient conversion of CS.

#### 3.1.2. Lower Temperatures

**Figure 4:**
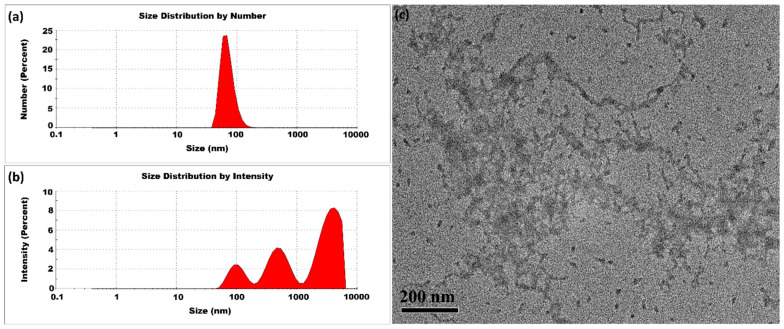
CSNPs synthesized at 10 °C with a CS:TPP ratio of 5:1. The final suspension was analyzed directly. (**a**) Size distribution by number percentage; (**b**) size distribution by intensity percentage. (**c**) TEM diagram.

The CSNPs synthesized at 10 °C had an average particle size of 509 ± 17 nm and a PDI of 1.00 ± 0.00, indicating substantial variability in particle size. The intensity profile shown in Figure 4 possessed three distinct peaks, suggesting the presence of multiple size populations. The moderate reduction in thermal energy slowed the molecular motion of both CS and TPP, which in turn reduced the frequency of ionic interactions. This slower interaction rate extended the curing phase and allowed larger aggregates to form. The higher PDI value come from a slower and more gradual crosslinking dependent on the moderated kinetic energy at 10 °C.

#### 3.1.3. High Temperatures

**Figure 5:**
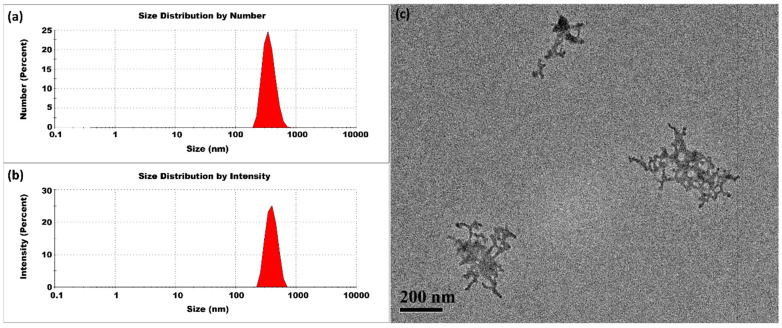
CSNPs synthesized at 40 °C with a CS:TPP ratio of 5:1. The final suspension was analyzed directly. (**a**) Size distribution by number percentage; (**b**) size distribution by intensity percentage. (**c**) TEM diagram.

The CSNPs synthesized at 40 °C had an average size of 429 ± 2 nm and a PDI of 0.359 ± 0.007. As shown in Figure 5, the size distribution analysis by number percentage indicated the presence of large-sized CSNPs, and the intensity percentage distribution also revealed a single peak, confirming the predominance of large-sized CSNPs in the sample. At 40 °C, the mobility of the initially formed small nuclei was significantly enhanced, leading to more frequent and effective collisions. This increased kinetic activity promotes the uniform aggregation of these small nuclei into larger CSNPs. These findings suggest that at 40 °C, the synthesis process may not provide enough thermal energy to fully overcome activation barriers, preventing complete curing of the small nuclei, but it does enable considerable curing. A temperature of 40 °C is sufficient to facilitate the formation of well-structured, large-sized CSNPs. This controlled aggregation effectively regulates the variance of the size, resulting in the formation of CSNPs with improved uniformity.

**Figure 6:**
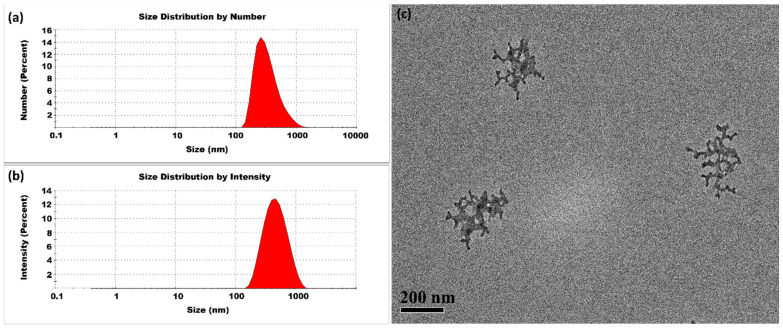
CSNPs synthesized at 50 °C with a CS:TPP ratio of 5:1. The final suspension was analyzed directly. (**a**) Size distribution by number percentage; (**b**) size distribution by intensity percentage. (**c**) TEM diagram.

Figure 6 shows results for CSNPs synthesized at 50 °C. The DLS and TEM analysis shown in Figure 6 indicates a notable change in the average particle size, with measurements of 370 ± 2 nm and PDI of 0.285 ± 0.001. At 50 °C, the curing process becomes more efficient than at 40 °C. This moderate improvement in curing efficiency at 50 °C reduces the tendency for multiple small nuclei to aggregate further.

**Figure 7:**
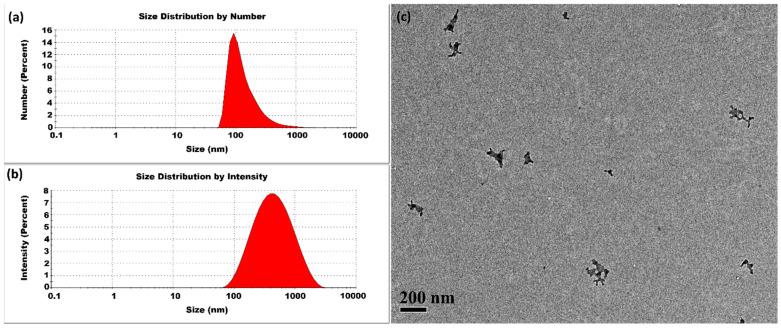
CSNPs synthesized at 80 °C with a CS:TPP ratio of 5:1. The final suspension was analyzed directly. (**a**) Size distribution by number percentage; (**b**) size distribution by intensity percentage. (**c**) TEM diagram.

The CSNPs synthesized at 80 °C showed an average particle size of 310 ± 2 nm, and a PDI value of 0.429 ± 0.008. The size distribution analysis by number percentage of DLS and TEM indicates the presence of small particles, as shown in Figure 7. At 80 °C, the rapid stabilization of small nuclei effectively curtails aggregations, leading to the decrease in nanoparticles size. The PDI of CSNPs significantly decreased from 0.956 under room temperature and 1.00 at 10 °C to 0.429 or lower at elevated temperature, highlighting the critical role of temperature in the curing process. The elevated temperature shortens the curing duration and reduces aggregation, resulting in the fabrication of small-sized CSNPs with a narrow size distribution.

**Figure 8:**
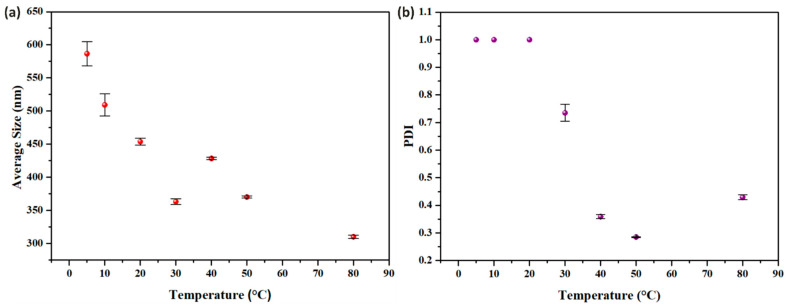
Impact of temperature on CSNPs: (**a**) Size, (**b**) PDI. The concentrations of CS and TPP are 2 mg/mL and 0.4 mg/mL, respectively. The final suspension was analyzed directly.

Figure 8 illustrates the dependence of average size and size distribution on temperature. At lower temperatures, the reduced Brownian motion, high activation barriers, slower reaction rates, and slow, inefficient, and uneven surface curing of small nuclei leads to uncontrollable aggregation, resulting in the formation of polydispersed CSNPs, as observed at 5 °C, 10 °C, 20 °C, and 30 °C. At 40 °C or higher temperatures, the increased Brownian motion promoted more frequent collisions between small nuclei, the activation barrier was well overcome to achieve a faster reaction rate and rapid cross-linking, and the small aggregates could remain largely independent, resulting in the stabilization of small-sized CSNPs with utmost uniformity. The abnormal increase in size from 30 °C to 40 °C might reflect the complex temperature effects on the aggregation (such as colloid probability and interface property).

In summary, the increasing temperature might simultaneously promote two factors: (1) particle surface curing and (2) particle collisions. The first factor, particle surface curing, acts to hinder agglomeration. When the effect of surface curing dominates, it can reduce agglomeration, which is a fundamental to produce CSNPs with utmost uniformity.

### 3.2. Impact of TPP Concentration on the Synthesis of CSNPs

**Figure 9:**
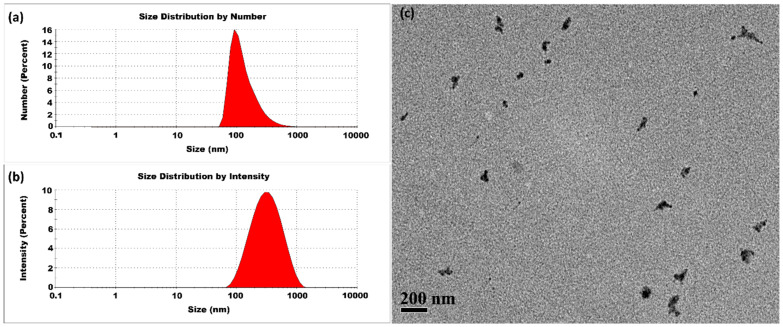
CSNPs synthesized at 80 °C with a CS:TPP ratio of 3.34:1. The final suspension was analyzed directly. (**a**) Size distribution by number percentage; (**b**) size distribution by intensity percentage. (**c**) TEM diagram.

The CSNPs were synthesized using a CS to TPP ratio of 3.34:1 at 80 °C, i.e., the TPP and CS concentrations were set at 0.6 mg/mL and 2 mg/mL, respectively. The products displayed an average particle size of 245 ± 1 nm and a PDI of 0.279 ± 0.007 (Figure 9). Compared to CSNPs synthesized using a 5:1 ratio, these results highlight a substantial reduction in particle average size and a decrease in the PDI. Since TPP functions as an ionic crosslinking agent to bind the amino groups on the CS molecules and form a gel-like matrix, the density of crosslinking sites will increase with the increasing of TPP concentration, thereby accelerating the curing process. This accelerated crosslinking facilitates the rapid formation of a more compact and rigid surface. Consequently, the aggregation of CSNPs will be more effectively regulated, preventing uncontrolled aggregation and size growth.

**Figure 10:**
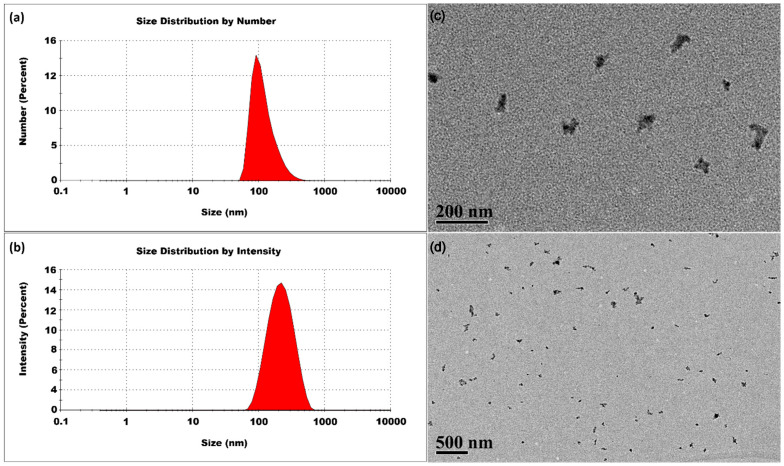
CSNPs synthesized at 80 °C with a CS:TPP ratio of 2.5:1. The final suspension was analyzed directly. (**a**) Size distribution by number percentage; (**b**) size distribution by intensity percentage; (**c**,**d**) TEM diagram.

Next, the CSNPs were synthesized by reacting CS with TPP at a 2.5:1 ratio and 80 °C. The concentrations of CS and TPP were 2 mg/mL and 0.6 mg/mL, respectively. As depicted in Figure 10, a decrease in the CS:TPP ratio from 3.34:1 to 2.5:1 caused a significant reduction in the average particle size, which decreased from 245 ± 1 nm to 188 ± 0.6 nm. Moreover, the PDI also improved, dropping from 0.279 ± 0.007 to 0.184 ± 0.004. The elevated TPP concentration significantly accelerates the crosslinking process, promoting the rapid formation of small nanoparticle nuclei within a very short period. This fast-paced crosslinking ensures that the nascent nuclei are stabilized almost immediately, preventing premature interactions between initially nucleated small nuclei. This early-stage stabilization is crucial for maintaining consistent particle size and preventing undesirable particle aggregation.

**Figure 11:**
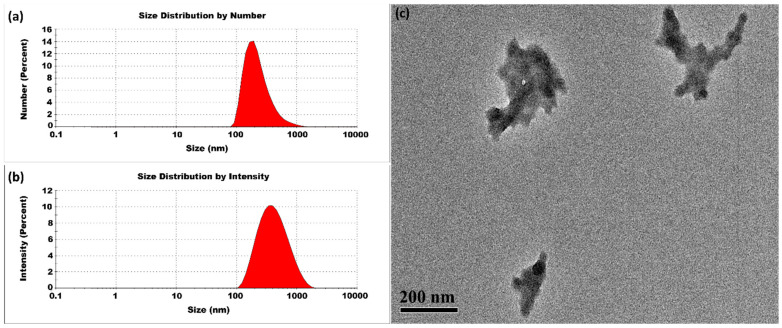
CSNPs synthesized at 80 °C with a CS:TPP ratio of 2:1. The final suspension was analyzed directly. (**a**) Size distribution by number percentage; (**b**) size distribution by intensity percentage. (**c**) TEM diagram.

When employing an even lower CS to TPP ratio, 2:1, moderate-sized CSNPs were synthesized instead of smaller ones as imagine. In detail, a CS concentration of 2 mg/mL and a TPP concentration of 1 mg/mL were used, resulting in CSNPs with an average size of 325 ± 0.7 nm and a PDI of 0.258 ± 0.007, as shown in Figure 11. We supposed that the excessive TPP content facilitated inter-crosslinking between the CSNPs, causing the nanoparticles to fuse together rather than remaining separate, leading to an increase in overall particle size.

**Figure 12:**
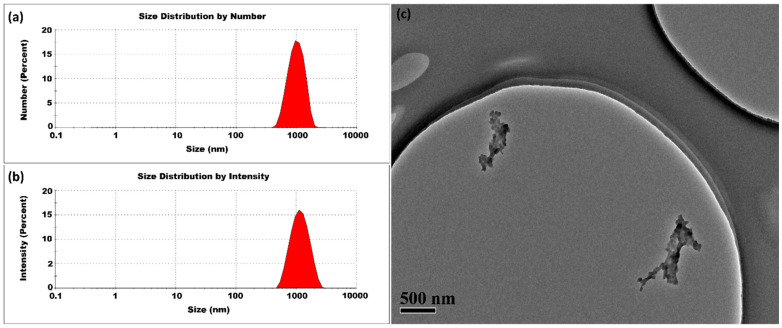
CSNPs synthesized at 80 °C with a CS:TPP ratio of 1.43:1. The final suspension was analyzed directly. (**a**) Size distribution by number percentage; (**b**) size distribution by intensity percentage. (**c**) TEM diagram.

When further decreasing the CS:TPP ratio to 1.43:1 (the concentrations of CS and TPP were 2 mg/mL and 1.4 mg/mL, respectively), we obtained even larger particles. The average size was 937 ± 8 nm and the PDI was 0.145 ± 0.03, respectively, as shown in Figure 12. Fortunately, the elevated temperature of 80 °C still played a crucial role in regulating the inter-crosslinking process, ultimately facilitating the production of relatively large-sized CSNPs with utmost uniformity.

**Figure 13:**
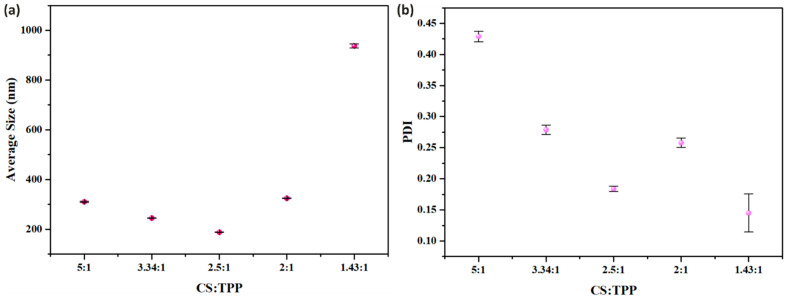
Impact of CS:TPP ratio on CSNPs: (**a**) particle size, (**b**) PDI. The concentration of CS is 2 mg/mL, and the temperature is 80 °C. The final suspension was analyzed directly.

Figure 13 demonstrates the influential role of TPP in governing the aggregation behavior of CSNPs. A CS:TPP ratio of 2.5:1 represents a critical threshold in CSNP: at this specific ratio, TPP efficiently facilitates crosslinking and stabilizes nascent nuclei within a short timeframe, yielding a highly uniform size distribution. As the ratio increases beyond this point, slower curing enhance the likelihood of continued interaction between nascent nuclei, even after they exit the delay tube, leading to uncontrolled aggregation and broad size dispersity. Conversely, decreasing the ratio below 2.5:1 intensifies interparticle crosslinking, promoting the formation of larger secondary aggregates.

The final suspensions of CSNPs prepared at 80 °C with CS:TPP ratios of 3.34:1, 2.5:1, and 2:1 exhibited stable colloidal behavior without turbidity or precipitation, indicating successful fabrication of CSNPs free of large aggregates or clusters. In contrast, at room temperature, the 3.34:1 ratio suspension showed turbidity and the 2:1 ratio suspension developed precipitation. These results highlight that elevated temperatures more effectively prevent uncontrollable aggregation, offering a superior approach to improve CSNP size uniformity and directly optimize target product yield.

Overall, this study demonstrates that high-temperature synthesis of CSNPs promotes more controlled nanoparticle growth compared to lower temperatures. CSNP aggregation is profoundly influenced by synthesis parameters, including microfluidic technology, temperature, and TPP concentration. Microfluidic systems enable rapid mixing and precise nucleation, generating small primary nuclei. At lower temperatures, slow and uneven crosslinking leads to insufficient stabilization, significantly increasing the likelihood of uncontrolled aggregation between nascent nuclei. Conversely, higher temperatures enhance molecular mobility, facilitating efficient crosslinking and shortening the surface-curing process of small nuclei. Optimizing surface curing under controlled collision rates allows precise regulation of CSNP aggregation and improves size uniformity. The synergistic effect of temperature and TPP is critical for enhancing CSNP stability and uniformity, ensuring the production of high-quality nanoparticles.

The influence of temperature on the acceleration of surface curing can be effectively correlated with the work of Shimiao Zhang et al. [68], who established a clear relationship between temperature and polymer surface curing. Their findings demonstrate that temperature plays a critical role in the curing process. At lower temperatures, the available thermal energy is insufficient to drive complete cross-linking, resulting in partial curing. In contrast, higher temperatures facilitate more extensive cross-linking, leading to a polymer network that approaches maximum density. This enhanced curing is achieved by gradually ramping the temperature to drive the reaction to completion and maximize cross-linking density [68].

Several theoretical models support our findings regarding the effect of temperature on the crosslinking process. According to the principles of Brownian motion, temperature directly influences the mobility of molecules; higher temperatures increase molecular movement, which can enhance crosslinking density. Fick’s First Law of Diffusion also indicates that the diffusion coefficient increases with temperature, promoting faster molecular transport and improving the crosslinking process. Additionally, the Arrhenius model demonstrates that elevated temperatures accelerate reaction rates by increasing the kinetic energy of molecules. In this context of CSNPs, higher temperatures enhance the reactivity of TPP, enabling it to more effectively optimize the crosslinking density and surface curing of CSNPs [69,70,71].

## 4. Conclusions

This study validates the effective synthesis of CSNPs via a microfluidic approach, where rapid mixing drives quick nucleation and formation of small nuclei. However, an initial high PDI of 0.956 ± 0.0239 at room temperature indicated substantial aggregation, with lower temperatures (10 °C and 5 °C) further increasing the polydispersity. High temperatures are pivotal for controlling CSNP growth by optimizing curing through regulated nuclear mobility, essential for fabricating highly uniform particles. At 40 °C and 50 °C, controlled interactions between small nuclei governed nanoparticle growth, yielding uniformly sized CSNPs with diameters of 429 ± 2 nm and 370 ± 2 nm, respectively. Raising the temperature to 80 °C drastically accelerated small-nucleus curing, resulting in smaller CSNPs. The CS:TPP ratio also critically influenced surface curing and particle size at 80 °C: ratios of 5:1, 3.34:1, and 2.5:1 produced CSNPs with mean sizes of 310 ± 2 nm, 245 ± 1 nm, and 188 ± 0.6 nm, respectively. Continuous decreases in both average size and PDI values reflected improved uniformity and reduced aggregation. The TPP-facilitated enhanced surface-curing process stabilized CSNPs early and minimized agglomeration, enabling successful synthesis of small CSNPs (188 ± 0.6 nm) at 80 °C with a 2.5:1 ratio. Additionally, precise size regulation at 80 °C yielded moderate and large CSNPs (325 ± 0.7 nm and 937 ± 8 nm, respectively). Collectively, temperature and TPP concentration are critical factors in modulating nanoparticle curing to control CSNP agglomeration. These parameters work in tandem to precisely regulate nucleus–nucleus interactions and particle–particle crosslinking, governing CSNP growth and size with exceptional consistency. This optimization enhances the translational potential of CSNPs for diverse applications.

## Data Availability

The original contributions presented in this study are included in the article/Supplementary Material. Further inquiries can be directed to the corresponding author.

## Supplementary Materials

The following supporting information can be downloaded at: https://www.mdpi.com/article/10.3390/mi16060642/s1.
