# Supplementary material for: Synthesis of Chitosan Nanoparticles via Microfluidic Approach: The Role of Temperature in Tailoring Aggregation for Enhanced Uniformity
Source: Micromachines (Basel). 2025 May 28;16(6):642. doi: 10.3390/mi16060642 (PMC12195486; doi:10.3390/mi16060642)
Supplement: Supplementary file 1 [file micromachines-16-00642-s001.zip › micromachines-3650089-supplementary.pdf]

Supporting Information

Synthesis of chitosan nanoparticles *via* microfluidic approach: The role of temperature in tailoring aggregation for enhanced uniformity

Muqarrab Ahmed[1], Yangcheng Lu[1*]

[1]State Key Laboratory of Chemical Engineering, Department of Chemical Engineering, Tsinghua University, Beijing 100084, China

[*]Corresponding author: luyc@tsinghua.edu.cn (Y. Lu)

Supporting Information contains the following content:

Table S1 CSNPs in drug delivery applications for cancer, diabetes, and tissue Engineering

Table S2 CSNPs in the treatment of viral diseases, including SARS-CoV-2, Influenza Virus, Newcastle Disease Virus, Human Immunodeficiency Virus, and Hepatitis

Table S3 CSNPs for optimization in antibacterial properties to treat several pathogens

Table S4 CSNPs in agricultural applications

Table S5 CSNPs in the separation process of metals and dyes

Table S6. CSNPs (5:1) at variable temperatures: mean size, mean PDI, SD, and SE.

Table S7. CSNPs (5:1) at variable CS: TPP ratio: mean size, mean PDI, SD, and SE.

Figure S1. Applied setup of microfluidic along with a delay tube for CSNPs fabrication

Figure S2. Experimental setup for measuring heat loss and optimizing pre-feed tube length to control temperature

Figure S3. Experimental setup for controlling high temperature using pre-heating tubes with a length of 300 cm

Figure S4. Experimental setup for controlling lower temperature using pre-cooling tubes with a length of 300 cm

Figure S5. Complete experimental setup for fabricating CSNPs

Figure S6. CSNPs synthesized at room temperature with a CS: TPP ratio of 5:1. The final suspension was analyzed directly. (a_1) Raw DLS curve showing Size distribution by number percentage; (a_2) Raw DLS curve showing Size distribution by intensity percentage. (b_1) Histogram showing Size distribution by number percentage; (b_2) Histogram showing Size distribution by intensity percentage.

Figure S7. CSNPs synthesized with a CS: TPP ratio of 5:1 at room temperature using a counter-flow microfluidic device. The samples were separated *via* centrifugation at 12,000 rpm for 30 minutes. Size of CSNPs separated in supernatant and size of aggregates in the pellet.

Figure S8. CSNPs synthesized at 10 °C with a CS: TPP ratio of 5:1. The final suspension was analyzed directly. (a1) Raw DLS curve showing Size distribution by number percentage; (a2) Raw DLS curve showing Size distribution by intensity percentage. (b1) Histogram showing Size distribution by number percentage; (b2) Histogram showing Size distribution by intensity percentage.

Figure S9. CSNPs synthesized at 40 °C with a CS: TPP ratio of 5:1. The final suspension was analyzed directly. (a1) Raw DLS curve showing Size distribution by number percentage; (a2) Raw DLS curve showing Size distribution by intensity percentage. (b1) Histogram showing Size distribution by number percentage; (b2) Histogram showing Size distribution by intensity percentage.

Figure S10. CSNPs synthesized at 50 °C with a CS: TPP ratio of 5:1. The final suspension was analyzed directly. (a1) Raw DLS curve showing Size distribution by number percentage; (a2) Raw DLS curve showing Size distribution by intensity percentage. (b1) Histogram showing Size distribution by number percentage; (b2) Histogram showing Size distribution by intensity percentage.

Figure S11. CSNPs synthesized at 80 °C with a CS: TPP ratio of 5:1. The final suspension was analyzed directly. (a1) Raw DLS curve showing Size distribution by number percentage; (a2) Raw DLS curve showing Size distribution by intensity percentage. (b1) Histogram showing Size distribution by number percentage; (b2) Histogram showing Size distribution by intensity percentage.

Figure S12. CSNPs with a CS: TPP ratio of 5:1, synthesized at 5 °C, 10 °C, 20 °C, 30 °C, 40 °C, 50 °C, and 80 °C using a counter flow microfluidic device, with replication performed three times. (a) Mean size ± SD; (b) SD; (c) Mean size ± SE; (d) SE.

Figure S13. CSNPs with a CS: TPP ratio of 5:1, synthesized at 5 °C, 10 °C, 20 °C, 30 °C, 40 °C, 50 °C, and 80 °C using a counter flow microfluidic device, with replication performed three times. (a) Mean PDI ± SD; (b) SD; (c) Mean PDI ± SE; (d) SE.

Figure S14. CSNPs synthesized at 80 °C with a CS: TPP ratio of 3.34:1. The final suspension was analyzed directly. (a1) Raw DLS curve showing Size distribution by number percentage; (a2) Raw DLS curve showing Size distribution by intensity percentage. (b1) Histogram showing Size distribution by number percentage; (b2) Histogram showing Size distribution by intensity percentage.

Figure S15. CSNPs with a CS: TPP ratio of 3.34: 1, synthesized at 80 °C using a microfluidic device. Obtained final suspension without any separation.

Figure S16. CSNPs synthesized at 80 °C with a CS: TPP ratio of 2.5:1. The final suspension was analyzed directly. (a1) Raw DLS curve showing Size distribution by number percentage; (a2) Raw DLS curve showing Size distribution by intensity percentage. (b1) Histogram showing Size distribution by number percentage; (b2) Histogram showing Size distribution by intensity percentage.

Figure S17. CSNPs synthesized at 80 °C with a CS: TPP ratio of 2:1. The final suspension was analyzed directly. (a1) Raw DLS curve showing Size distribution by number percentage; (a2) Raw DLS curve showing Size distribution by intensity percentage. (b1) Histogram showing Size distribution by number percentage; (b2) Histogram showing Size distribution by intensity percentage.

Figure S18. CSNPs synthesized at 80 °C with a CS: TPP ratio of 1.43:1. The final suspension was analyzed directly. (a1) Raw DLS curve showing Size distribution by number percentage; (a2) Raw DLS curve showing Size distribution by intensity percentage. (b1) Histogram showing Size distribution by number percentage; (b2) Histogram showing Size distribution by intensity percentage.

Figure S19. CSNPs with a CS: TPP ratio of 5:1, 3.34:1, 2.5:1, 2:1, and 1.43:1, synthesized at 80 °C using a counter flow microfluidic device, with replication performed three times. (a) Mean size ± SD; (b) SD; (c) Mean size ± SE; (d) SE.

Figure S20. CSNPs with a CS: TPP ratio of 5:1, 3.34:1, 2.5:1, 2:1, and 1.43:1, synthesized at 80 °C using a counter flow microfluidic device, with replication performed three times. (a) Mean PDI ± SD; (b) SD; (c) Mean PDI ± SE; (d) SE.

Figure S21. Representation of Size vs. Temperature with exponential fit

Chitosan Nanoparticles Potential Applications and Size range

Table S1 CSNPs in drug delivery applications for cancer, diabetes, and tissue Engineering

Serial No.	CSNPs size	Applications in biomedical filed	Drug delivery	Reference
1	100-180 nm	Chemotherapy for cancer treatment	Delivery of anti-tumor agent 5-Fluorouracil *via* CSNPs	[1]
2	180-200 nm	Chemotherapy for cancer treatment	Delivery of anti-tumor agent curcumin *via* CSNPs	[2]
3	250-350 nm	Optical/MR dual imaging for cancer treatment	Delivery of Cy5.5 Fluorescent Dye *via* CSNPs	[3]
4	180-200 nm	Photo-thermal therapy for cancer treatment	Delivery of photo-thermal agents IR780 *via* CSNPs	[4]
5	250-300 nm	Chemotherapy for cancer treatment	Delivery of anti-tumor agent carboplatin *via* CSNPs	[5]
6	186-250 nm	Treatment of diabetes mellitus type II	Delivery of anti-diabetic agent sitagliptin *via* CSNPs	[6]
7	270-670 nm	Treatment of diabetes: Pr e-diabetic levels	Delivery of anti-diabetic agent insulin *via* CSNPs	[7]
8	56-144 nm	Treatment of diabetes type 2 diabetes therapy	Delivery of anti-diabetic agent polydatin *via* CSNPs	[8]
9	100-300 nm	Treatment of the tissue repairing and engineering	Delivery of plasmid DNA *via* CSNPs	[9]
10	180-250 nm	Treatment of the tissue repairing and engineering	Delivery of siRNA *via* CSNPs	[10]

Table S2 CSNPs in the treatment of viral diseases, including SARS-CoV-2, Influenza Virus, Newcastle Disease Virus, Human Immunodeficiency Virus, and Hepatitis

Serial No.	CSNPs Size	Viral disease	Delivery procedure	Reference
1	150-200 nm	Treatment of viral infection SARS-CoV-2	Anti-viral oral drug delivery *via* CSNPs	[11]
2	180-290 nm	Treatment of viral infection SARS-CoV-2	Subunit vaccine delivery *via* CSNPs	[12]

3	200-300 nm	Treatment of viral infection Influenza Virus	Influenza vaccines delivery *via* CSNPs	[13]
4	179-358 nm	Treatment of viral infection Influenza virus	Hemagglutinin-Split Influenza Virus Mucosal Vaccine *via* CSNPs	[14]
5	153-200 nm	Treatment of viral infection Swine Influenza Virus	Swine influenza DNA vaccine delivery *via* CSNPs	[15]
6	323-571 nm	Treatment of viral infection Swine influenza A	Inactivated SwIAV vaccine delivery *via* CSNPs	[16]
7	371-500 nm	Treatment of viral infection Newcastle Disease Virus NDV	Inactivated ND vaccine delivery *via* CSNPs	[17]
8	212-342 nm	Treatment of viral infection Human immunodeficiency virus (HIV)	Therapeutic antigen HIV delivery *via* CSNPs	[18]
9	380-500 nm	Treatment of viral infection Dengue viruses (DENVs)	Novel dengue nano-vaccine (DNV) delivery *via* CSNPs	[19]
10	160-200 nm	Treatment of viral infection Hepatitis Viral Infection	Anti-hepatitis vaccine delivery *via* CSNPs	[20]

Table S3 CSNPs for optimization in antibacterial properties to treat several pathogens

Serial No.	CSNPs Size	Pathogens	Applications	Reference
1	200-350 nm	Inhibition of the pathogens such as Escherichia coli and Staphylococcus aureus	Optimization in the bioavailability and stability of the anti-bacterial agent like curcumin *via* CSNPs in gastric and intestinal digestion	[21]
2	180-280 nm	Inhibition of the pathogens such as Morganella morganii and Escherichia coli	Optimization in the coating preservation of salmon fillets. approach to extend the shelf life of food	[22]
3	160-280 nm	Inhibition of the pathogens such as Escherichia coli and Staphylococcus aureus	Optimization in the stability of quercetin *via* CSNPs in industrial food production	[23]

4	150-180 nm	Inhibition of the pathogens such as Escherichia coli and Staphylococcus aureus	Food preservation of fresh pork by enhancing stability and prolong bioactivity *via* CSNPs	[24]
5	121-152 nm	Inhibition of the pathogens such as Escherichia coli and Staphylococcus aureus	Optimization in the bioavailability and stability of the anti-bacterial agent like curcumin *via* CSNPs for wound dressing	[25]
6	122-140 nm	Inhibition of the pathogens such as V. cholera and C. jejuni	Optimization in the bioavailability and stability of the anti-bacterial agent like selenium *via* CSNPs	[26]
7	330-480 nm	Inhibition of the pathogens such as Escherichia coli and Staphylococcus aureus	Optimization in the stability and antibacterial properties of the Hydrastis canadensis *via* CSNPs	[27]
8	200-280 nm	Inhibition of the pathogens such as Escherichia coli and Staphylococcus aureus	Optimization in the anti-bacterial properties of the Eucommia ulmoides essential oil *via* CSNPs	[28]
9	170-380 nm	Inhibition of the pathogens such as Escherichia coli and Candida albicans	Optimization in the vaginal admiration *via* CSNPs for the treatment of Bacterial vaginosis (BV) chronic infection	[29]
10	130-180 nm	Inhibition of the pathogens such as C. tropicalis and C. parapsilosis	Optimization in the stability and antibacterial properties of bioactive molecules *via* CSNPs	[30]

Table S4 CSNPs in agricultural applications

Serial No.	CSNPs Size	Potential Application	Function	Reference
1	200-260 nm	Seed treatment	Optimization in the growth of wheat (Triticum aestivum L.) seeds *via* C SNPs	[31]
2	400-500 nm	Seed treatment	Optimization in the seed germination such as maize seeds *via* CSNPs	[32]

3	160-380 nm	Seed treatment	Optimization in the seed germination such as maize seeds *via* CSNPs	[33]
4	172-352 nm	Seed treatment	Optimization in the growth of the wheat (Triticum vulgare), oat (Avena sativa), and barley (Hordeum vulgare) seeds *via* CSNPs	[34]
5	40-50 nm	Soil treatment	Optimization in the process of removing contaminants from soil like Cadmium (Cd) polluted soils *via* CSNPs	[35]
6	200-500 nm	Soil treatment	Combating crop pathogens, minimizing nutrient losses in fertilization *via* CSNPs for improving crop bioavailability productivity	[36]

Table S5 CSNPs in the separation process of metals and dyes

Serial No.	Size	Potential Application	Function	Reference
1	100-320 nm	Metals removal from Industrial Waste Water	Optimization in the removal process of Fe (II) and Mn (II) *via* CSNPs	[37]
2	50-100 nm	Metals removal from Industrial Waste Water	Optimization in the removal process of Pb^{2+} , $Cd2^{+}$ and Zn^{2+} *via* CSNPs	[38]
3	100-180 nm	Dyes removal from industrial waste water	Optimization in the removal process of Acid Green 27 (AG27) dye *via* CSNPs	[39]
4	240-650 nm	Dyes removal from industrial waste water	Optimization in the removal process of Anionic phthalocyanine dye *via* CSNPs	[40]
5	80-250 nm	Dyes removal from industrial waste water	Optimization in the removal process of Reactive Red 120 (RR120) dye *via* CSNPs	[41]

Experimental setup of microfluidics along with a delay tube

The applied microfluidic device (i.d. = 0.5 mm) and delay tube (i.d. = 2 mm, length = 637 cm) for the fabrication of CSNPs, shown in Figure S1.

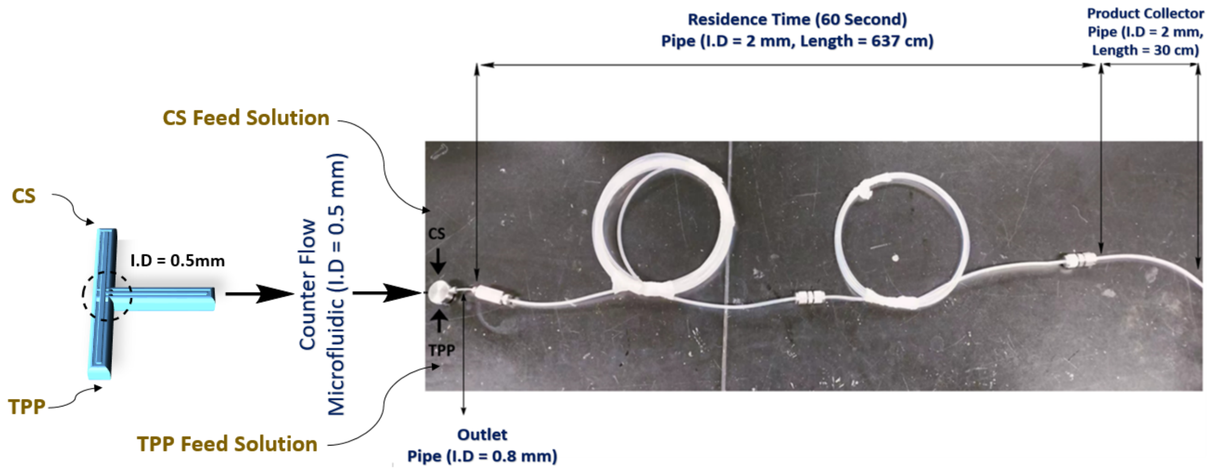

Figure S1. Applied setup of microfluidic along with a delay tube for CSNPs fabrication

Selection of optimum pre-feed tube dimensions

This setup allowed us to observe and measure the heat loss during the process. Two different tube lengths (100 cm and 300 cm) having internal diameter of 0.8 mm were tested to examine the effects of tube size on the temperature control deviation. During the tests, the water temperature was continuously monitored at the endpoint of the tubes to ensure accurate tracking of the heat transfer process. A multi-temperature recorder was used to measure temperature. A 100 cm tube showed a little temperature deviation, while no temperature deviation was detected in the 300 cm pre-feed tube, shown in Figure S2.

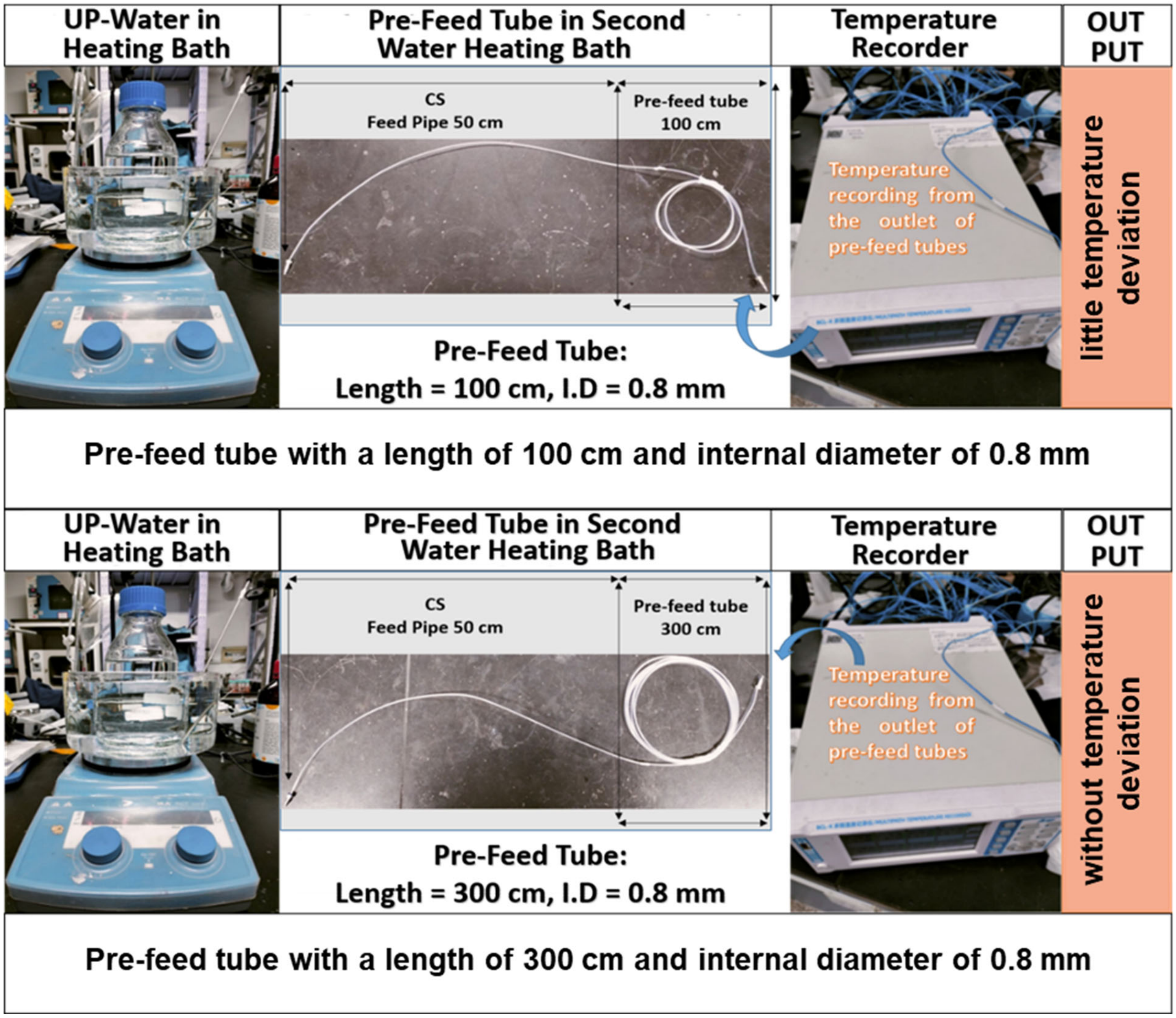

Figure S2. Experimental setup for measuring temperature deviation and optimizing pre-feed tube length to control temperature

Temperature-controlled pre-heated feed solutions for CS and TPP

The optimum pre-feed tube length of 300 cm was applied to control the temperature of the pre-heated CS and TPP feed solutions. To measure heat loss from the CS and TPP feed solution from preheating tube of 300 cm, an advanced water heating bath equipped with stirring and temperature control was employed. These water heating baths provided a sufficient depth of water surrounding the bottles containing CS and TPP, creating a more controlled environment for accurate temperature measurements. The bottle was properly immersed in the heated water bath to ensure uniform heating. Two separate heating baths were used for the CS and TPP feed solutions, while

the pre-heating tubes were immersed in a third heating bath. We used a multi-temperature detector to verify the high temperatures of the CS and TPP feed solutions at the outlet, which demonstrated that the high temperatures were successfully controlled, shown in Figure S3.

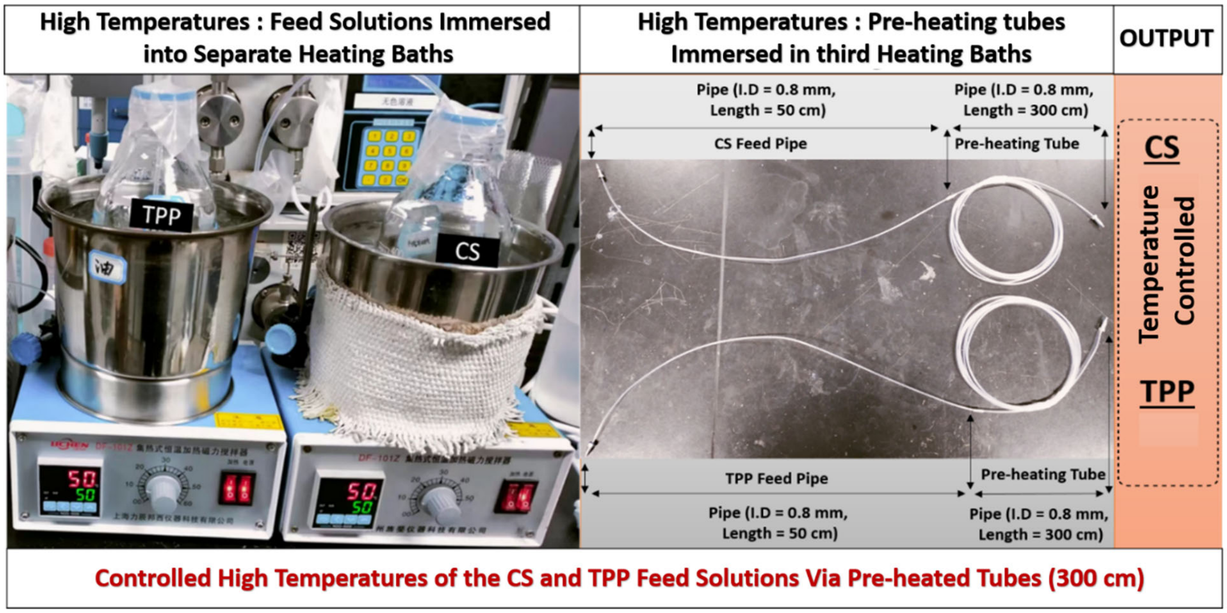

Figure S3. Experimental setup for controlling high temperature using pre-heating tubes with a length of 300 cm

Temperature-controlled pre-cooled feed solutions for CS and TPP

To control the temperature of the pre-cooled CS and TPP feed solutions, a distinct approach was adopted. This pre-cooling process focused on maintaining the feed solutions at a consistently low temperature, requiring a more rigorous cooling mechanism. The CS and TPP feed solutions, along with the pre-cooling tubes (each 300 cm in length), were placed in an advanced ethanol-based cooling bath designed to stabilize the temperature of the feed solutions effectively. The cooling bath, equipped with precise temperature control and circulation capabilities, ensured continuous and uniform cooling of the solutions by circulating the cooling medium around the tubes. We used a multi-temperature detector to verify the lower temperatures of the CS and TPP feed solutions at the outlet, which demonstrated that the lower temperatures were successfully controlled, shown in Figure S4.

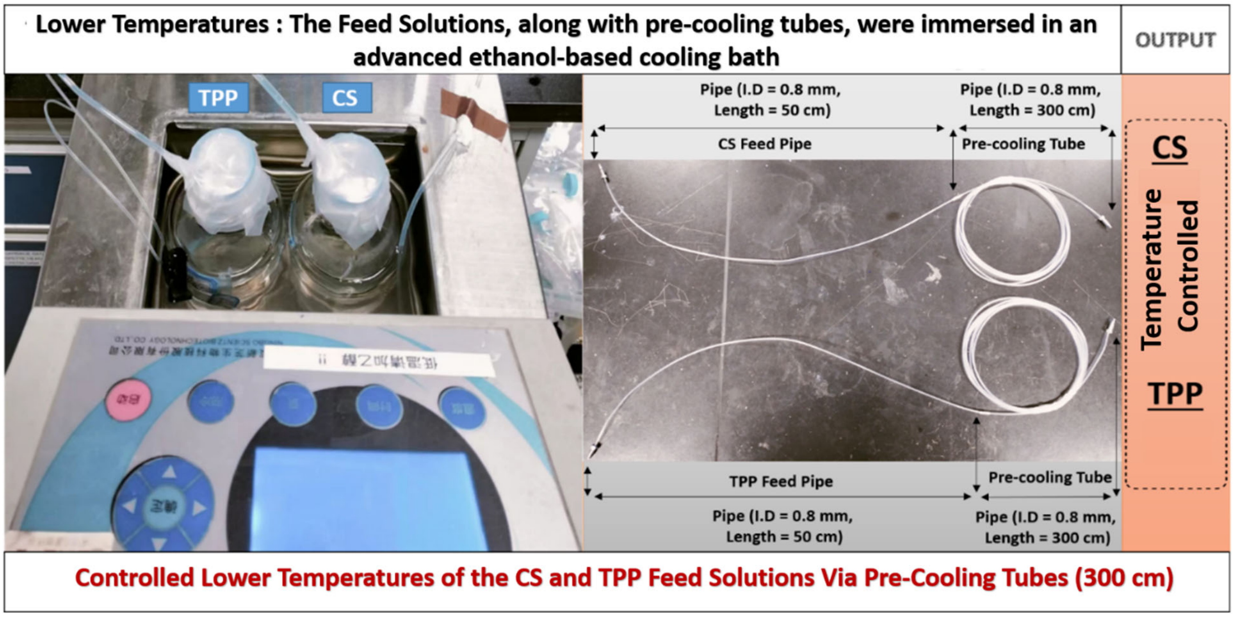

Figure S4. Experimental setup for controlling lower temperature using pre-feed tubes with a length of 300 cm

Complete operational setup

The diagram below shows the complete operational system used for the fabrication of CSNPs. To control the temperature, this entire experimental setup involved the immersion of the following components in a thermostat: feed bottles, 300 cm pre-cooling and pre-heating tubes, the micro-mixer, and the delay tube, shown in Figure S5.

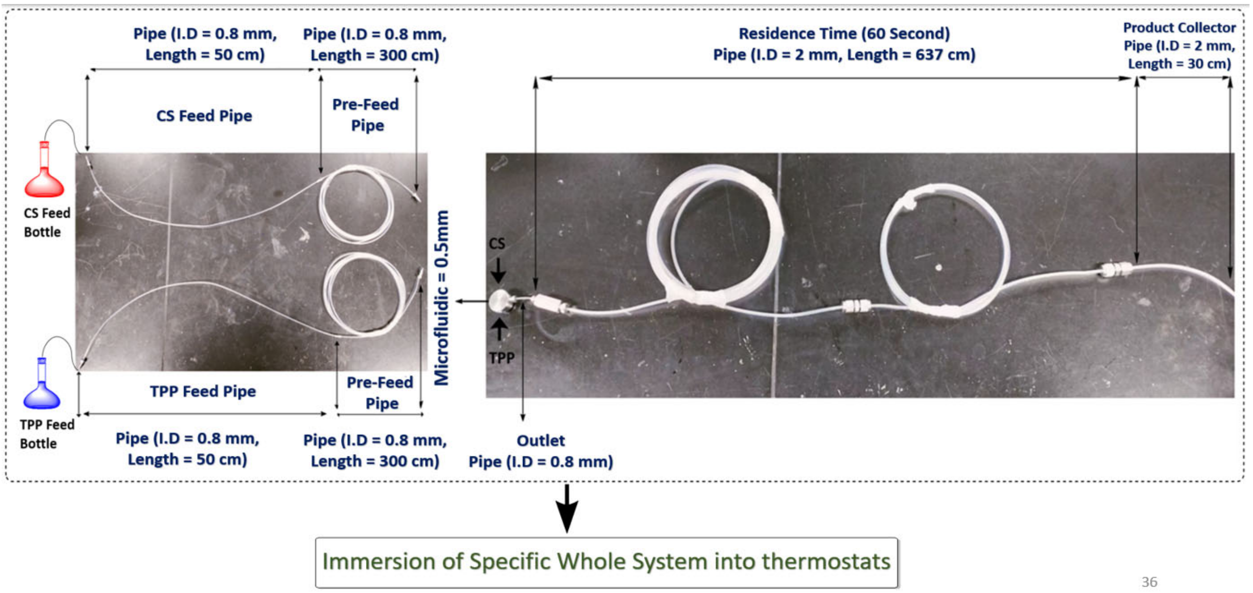

Figure S5. Complete experimental setup for fabricating CSNPs

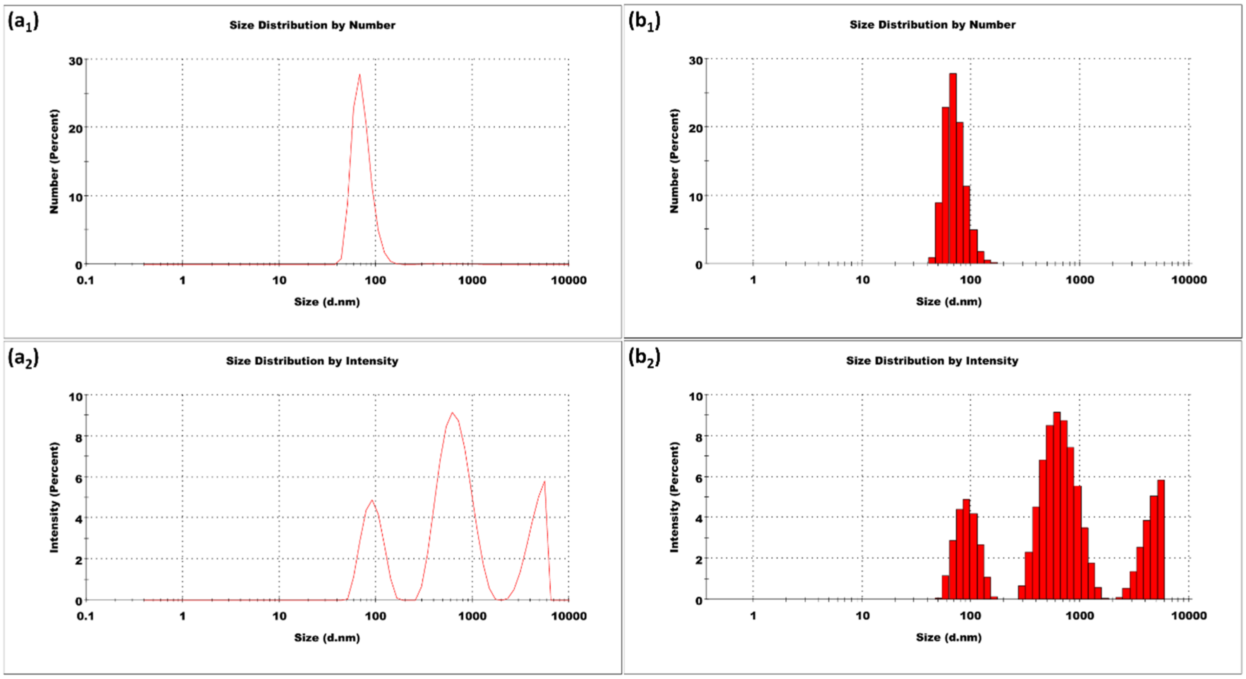

Figure S6. CSNPs synthesized at room temperature with a CS: TPP ratio of 5:1. The final suspension was analyzed directly. (a_1) Raw DLS curve showing Size distribution by number percentage; (a_2) Raw DLS curve showing Size distribution by intensity percentage. (b_1) Histogram showing Size distribution by number percentage; (b_2) Histogram showing Size distribution by intensity percentage.

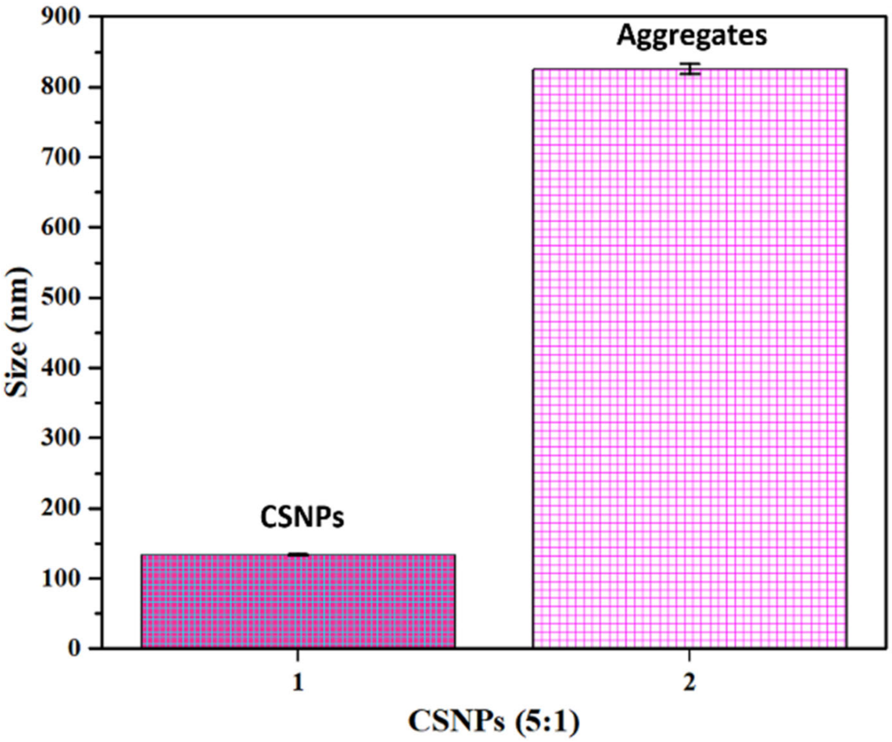

Figure S7. CSNPs synthesized with a CS:TPP ratio of 5:1 at room temperature using a counter-flow microfluidic device. The samples were separated *via* centrifugation at 12,000 rpm for 30 minutes. Size of CSNPs separated in supernatant and size of aggregates in the pellet.

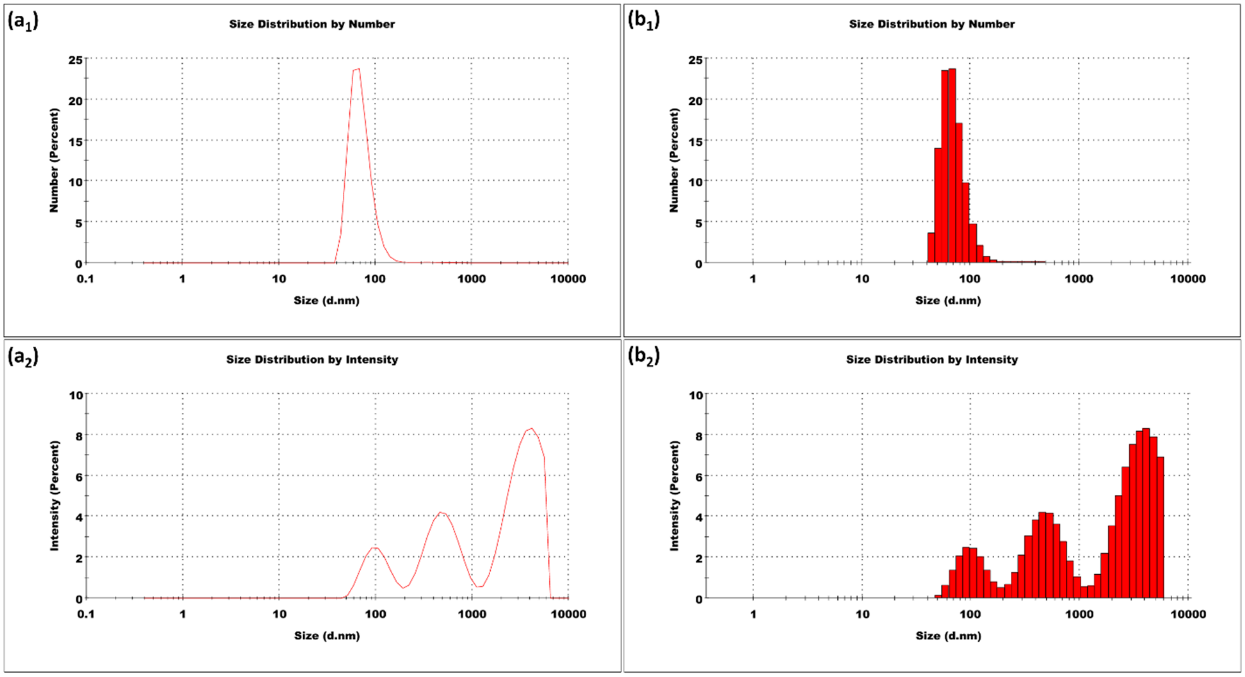

Figure S8. CSNPs synthesized at 10 °C with a CS: TPP ratio of 5:1. The final suspension was analyzed directly. (a1) Raw DLS curve showing Size distribution by number percentage; (a2) Raw DLS curve showing Size distribution by intensity percentage. (b1) Histogram showing Size distribution by number percentage; (b2) Histogram showing Size distribution by intensity percentage.

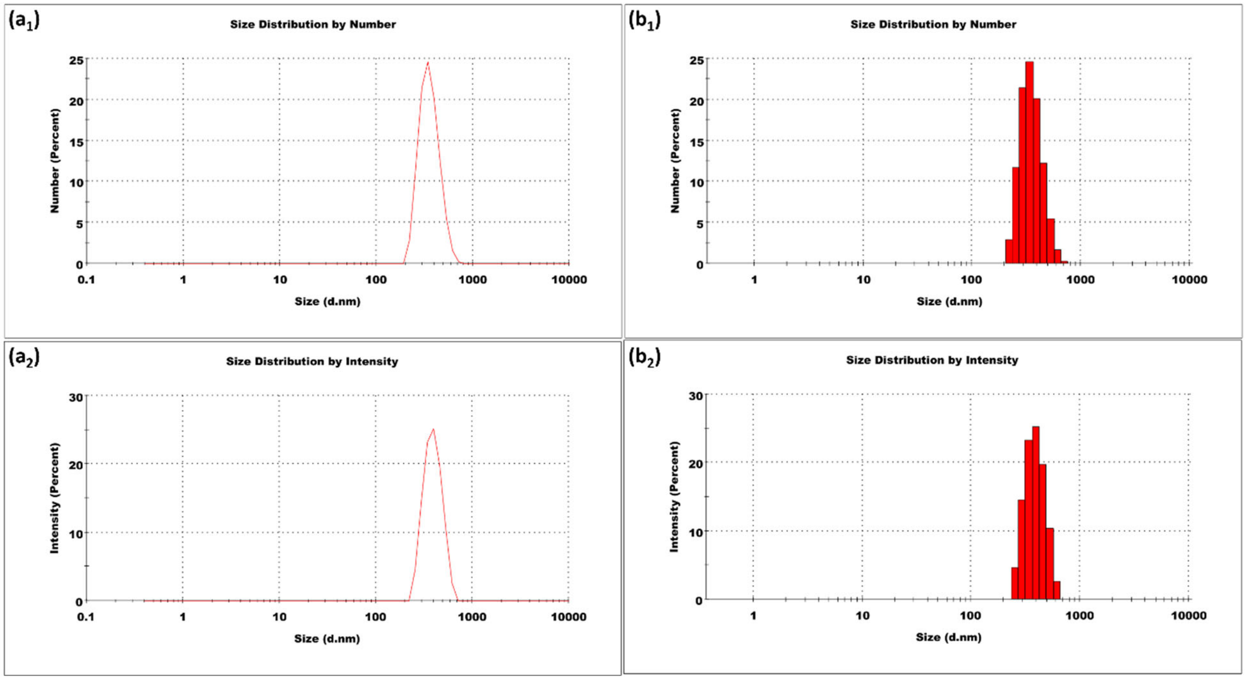

Figure S9. CSNPs synthesized at 40 °C with a CS: TPP ratio of 5:1. The final suspension was analyzed directly. (a1) Raw DLS curve showing Size distribution by number percentage; (a2) Raw DLS curve showing Size distribution by intensity percentage. (b1) Histogram showing Size distribution by number percentage; (b2) Histogram showing Size distribution by intensity percentage.

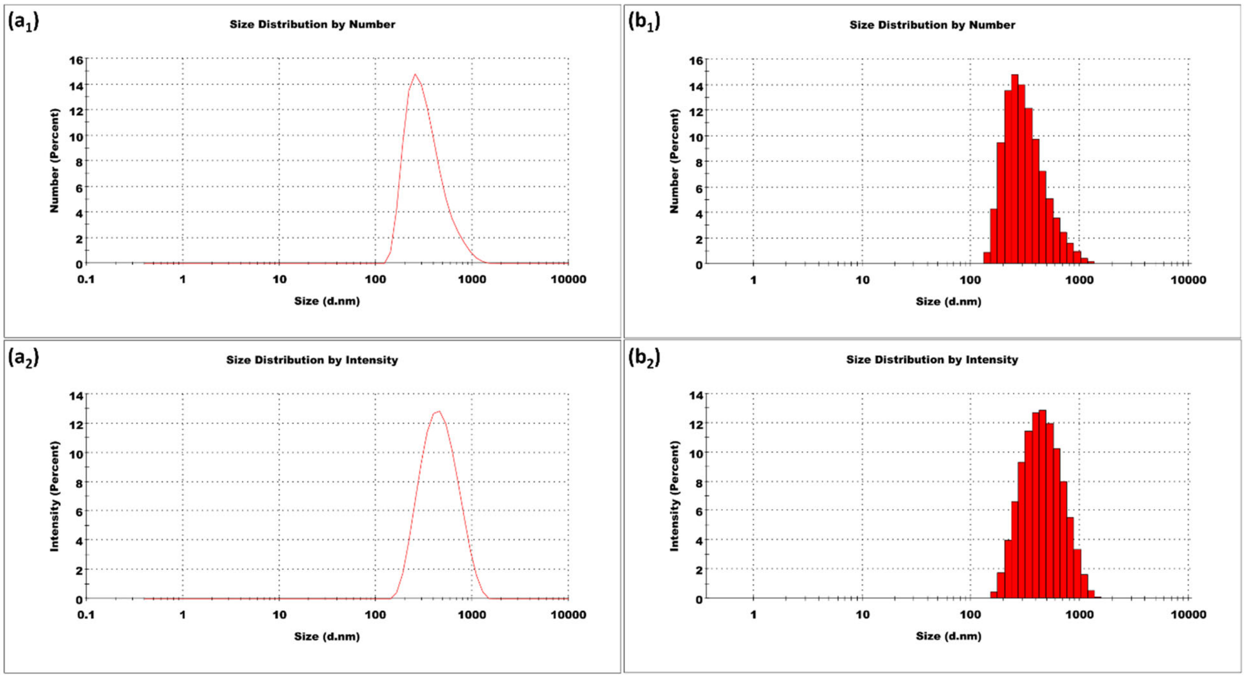

Figure S10. CSNPs synthesized at 50 °C with a CS: TPP ratio of 5:1. The final suspension was analyzed directly. (a1) Raw DLS curve showing Size distribution by number percentage; (a2) Raw DLS curve showing Size distribution by intensity percentage. (b1) Histogram showing Size distribution by number percentage; (b2) Histogram showing Size distribution by intensity percentage.

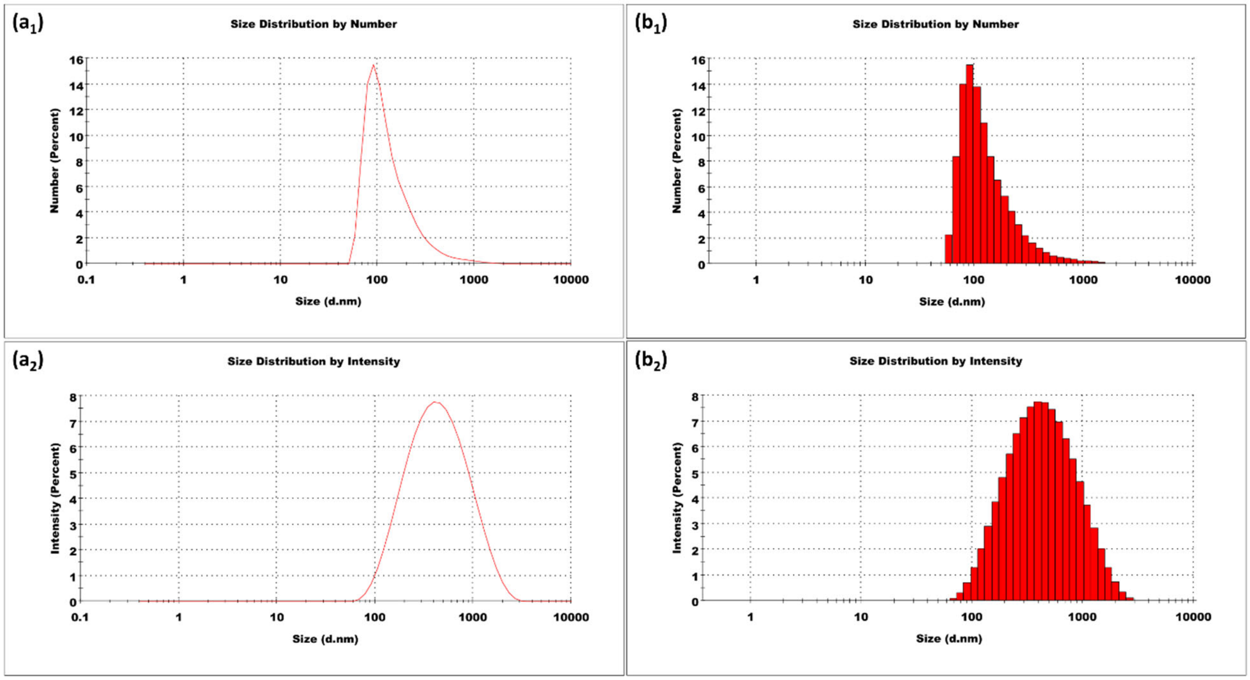

Figure S11. CSNPs synthesized at 80 °C with a CS: TPP ratio of 5:1. The final suspension was analyzed directly. (a1) Raw DLS curve showing Size distribution by number percentage; (a2) Raw DLS curve showing Size distribution by intensity percentage. (b1) Histogram showing Size distribution by number percentage; (b2) Histogram showing Size distribution by intensity percentage.

Table S6. CSNPs (5:1) at variable temperatures: mean size, mean PDI, SD, and SE.

T (°C)	CS $\frac{mg}{mL}$	TPP $\frac{mg}{mL}$	CS:TPP	Size (nm) xi	Mean (nm) $\bar{x} = \frac{1}{n}\sum_{i=1}^{n} x$	SD $\frac{\sqrt{(xi-\bar{x})}}{n-1}$	SE $\frac{SD}{\sqrt{n}}$	PDI	Mean (nm) $\bar{x} = \frac{1}{n}\sum_{i=1}^{n} x$	SD $\frac{\sqrt{(xi-\bar{x})^2}}{n-1}$	SE $\frac{SD}{\sqrt{n}}$
5	2	0.4	5:1	558<br>621<br>581	586.6	±31.87	±18.4	1.00<br>1.00<br>1.00	1.00	±0.00	±0.00
10	2	0.4	5:1	540<br>505<br>483	509.3	±28.74	±16.6	1.00<br>1.00<br>1.00	1.00	±0.00	±0.00
20	2	0.4	5:1	450<br>464<br>448	454	±8.71	±5.03	1.00<br>1.00<br>1.00	1.00	±0.00	±0
30	2	0.4	5:1	371<br>363<br>356	363.3	±7.50	±4.33	0.710<br>0.700<br>0.797	0.735	±0.0533	±0.0308
40	2	0.4	5:1	431<br>425<br>430	428.6	±3.21	±1.85	0.346<br>0.368<br>0.364	0.359	±0.0117	±0.0067
50	2	0.4	5:1	371<br>367<br>373	370.3	±3.055	±1.76	0.285<br>0.283<br>0.288	0.285	±0.0025	±0.0014
80	2	0.4	5:1	313<br>312<br>306	310.3	±3.78	±2.18	0.412<br>0.439<br>0.436	0.429	±0.0147	±0.0085

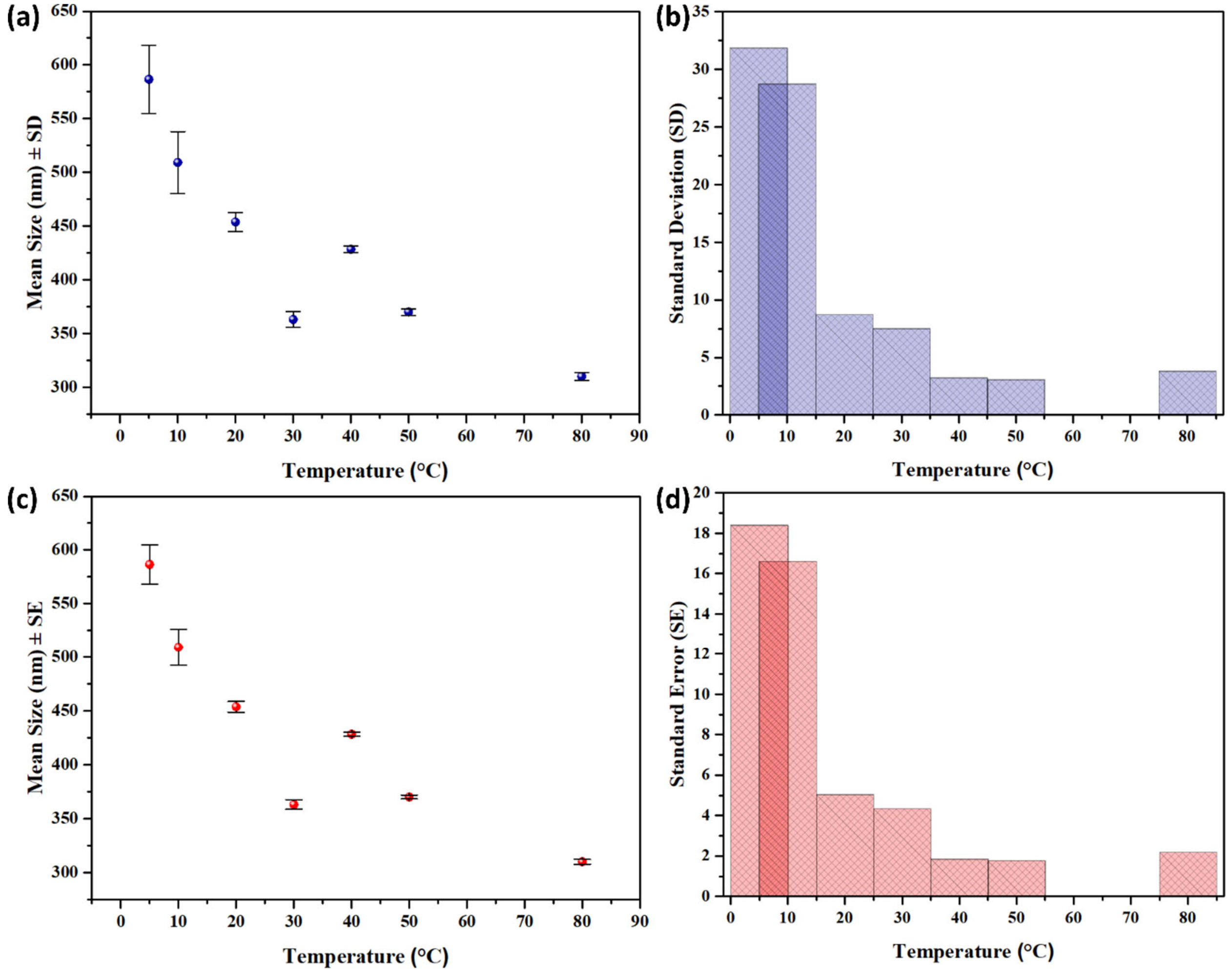

Figure S12. CSNPs with a CS: TPP ratio of 5:1, synthesized at 5 °C, 10 °C, 20 °C, 30 °C, 40 °C, 50 °C, and 80 °C using a counter flow microfluidic device, with replication performed three times. (a) Mean size ± SD; (b) SD; (c) Mean size ± SE; (d) SE.

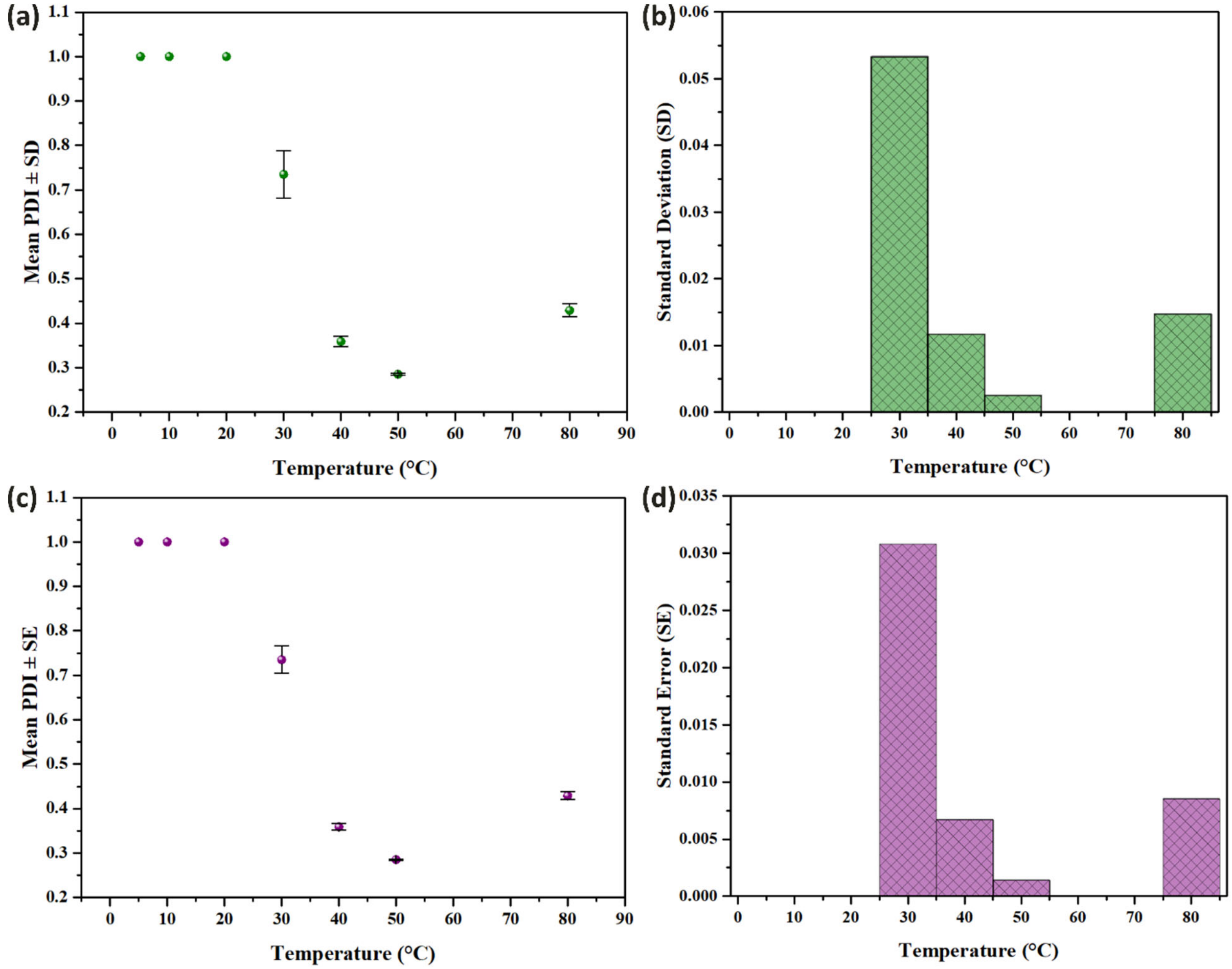

Figure S13. CSNPs with a CS: TPP ratio of 5:1, synthesized at 5 °C, 10 °C, 20 °C, 30 °C, 40 °C, 50 °C, and 80 °C using a counter flow microfluidic device, with replication performed three times. (a) Mean PDI ± SD; (b) SD; (c) Mean PDI ± SE; (d) SE.

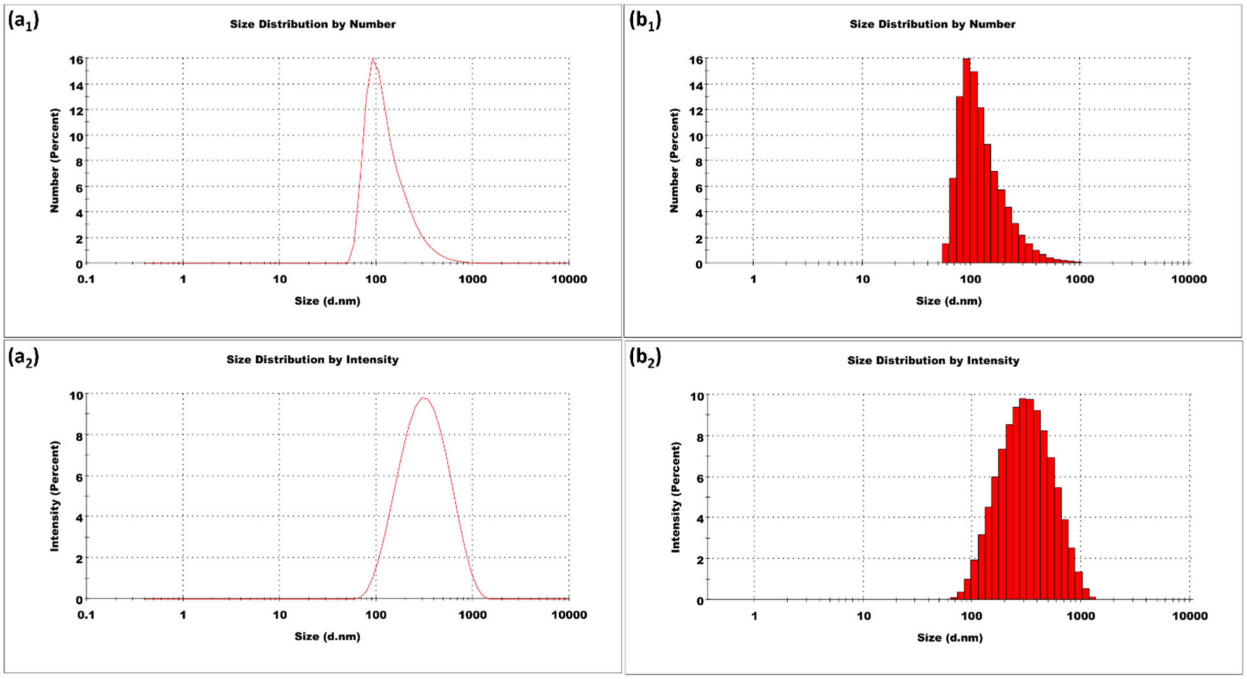

Figure S14. CSNPs synthesized at 80 °C with a CS: TPP ratio of 3.34:1. The final suspension was analyzed directly. (a1) Raw DLS curve showing Size distribution by number percentage; (a2) Raw DLS curve showing Size distribution by intensity percentage. (b1) Histogram showing Size distribution by number percentage; (b2) Histogram showing Size distribution by intensity percentage.

Fabrication of CSNPs at elevated temperature of 80 °C

The CSNPs were synthesized *via* microfluidic using a CS concentration of 2 mg/mL and TPP at 0.6 mg/mL, resulting in a CS: TPP ratio of 3.34:1. The final CSNPs suspension, prepared with a 3.34:1 CS: TPP ratio at 80 °C, is a clear colloidal suspension, free from turbidity or precipitation, shown in **Figure S6**.

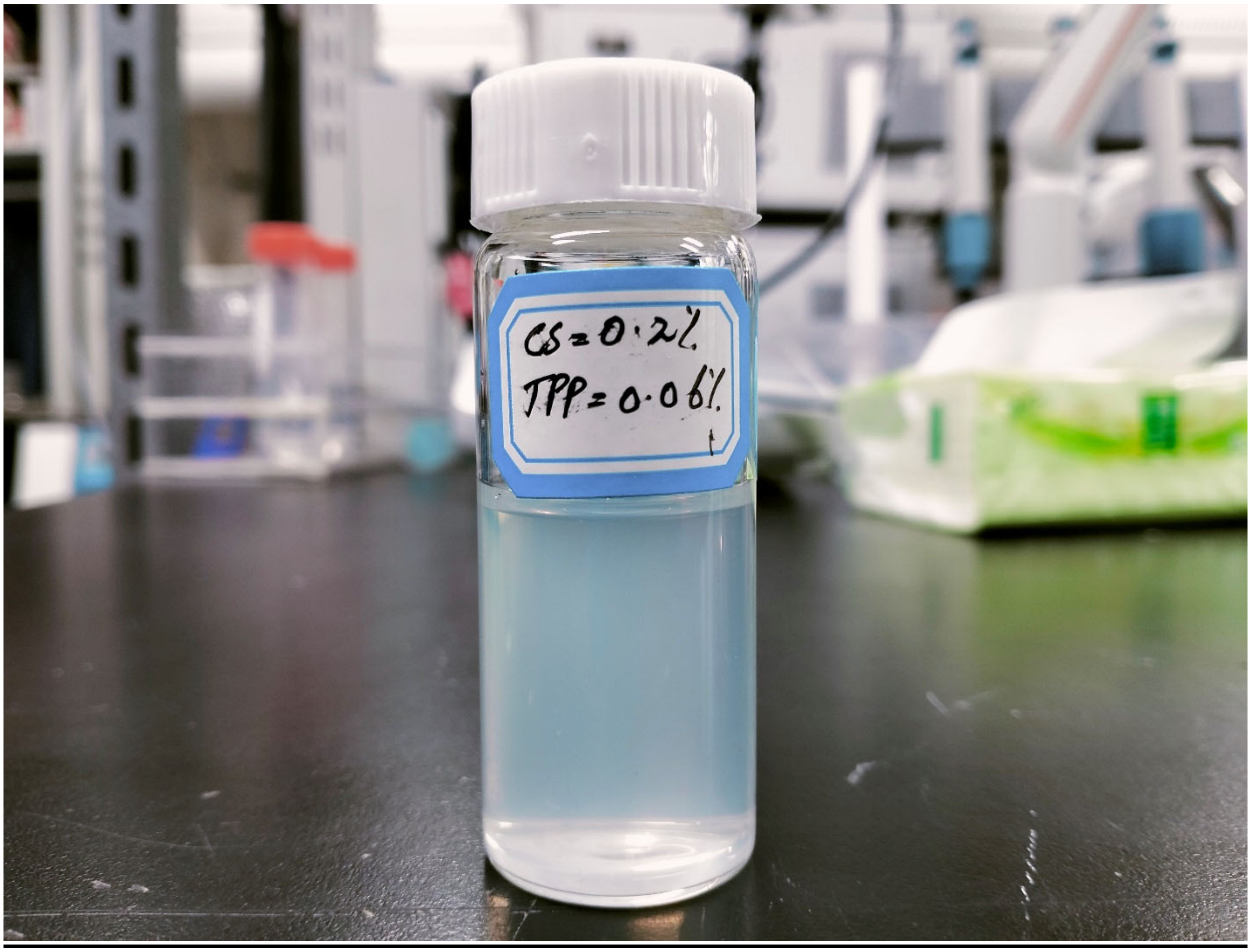

Figure S15. CSNPs with a CS: TPP ratio of 3.34: 1, synthesized at 80 °C using a microfluidic device. Obtained final suspension without any separation.

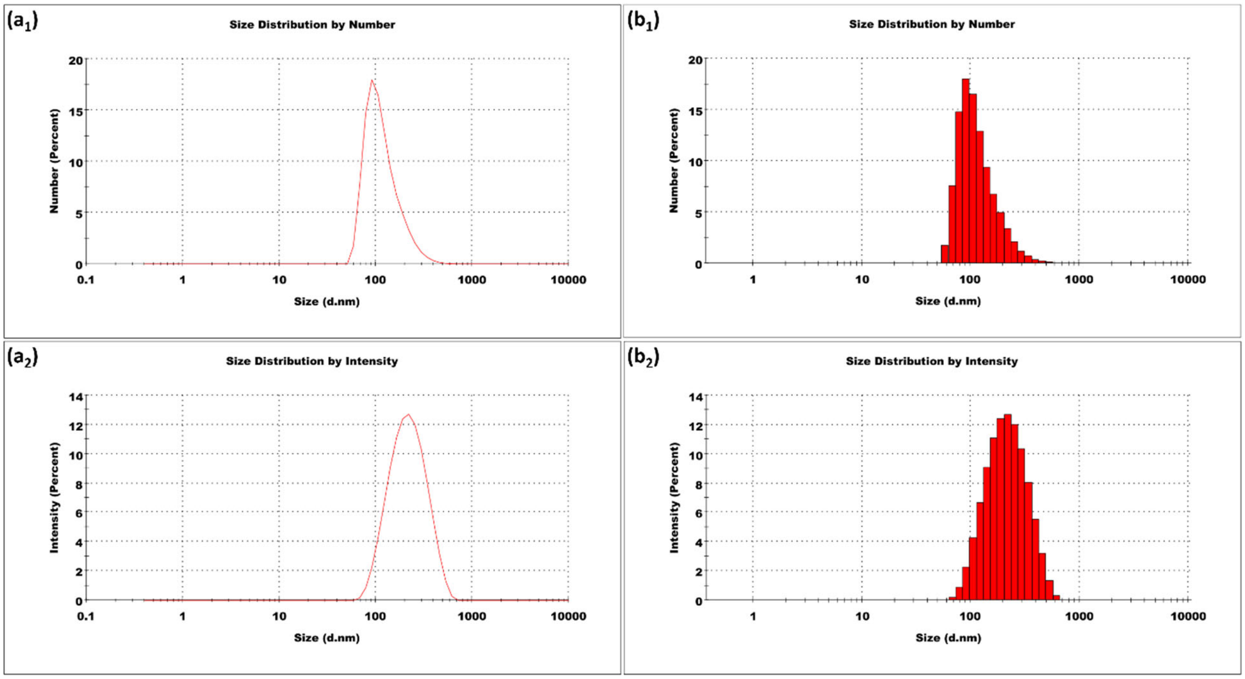

Figure S16. CSNPs synthesized at 80 °C with a CS: TPP ratio of 2.5:1. The final suspension was analyzed directly. (a1) Raw DLS curve showing Size distribution by number percentage; (a2) Raw DLS curve showing Size distribution by intensity percentage. (b1) Histogram showing Size distribution by number percentage; (b2) Histogram showing Size distribution by intensity percentage.

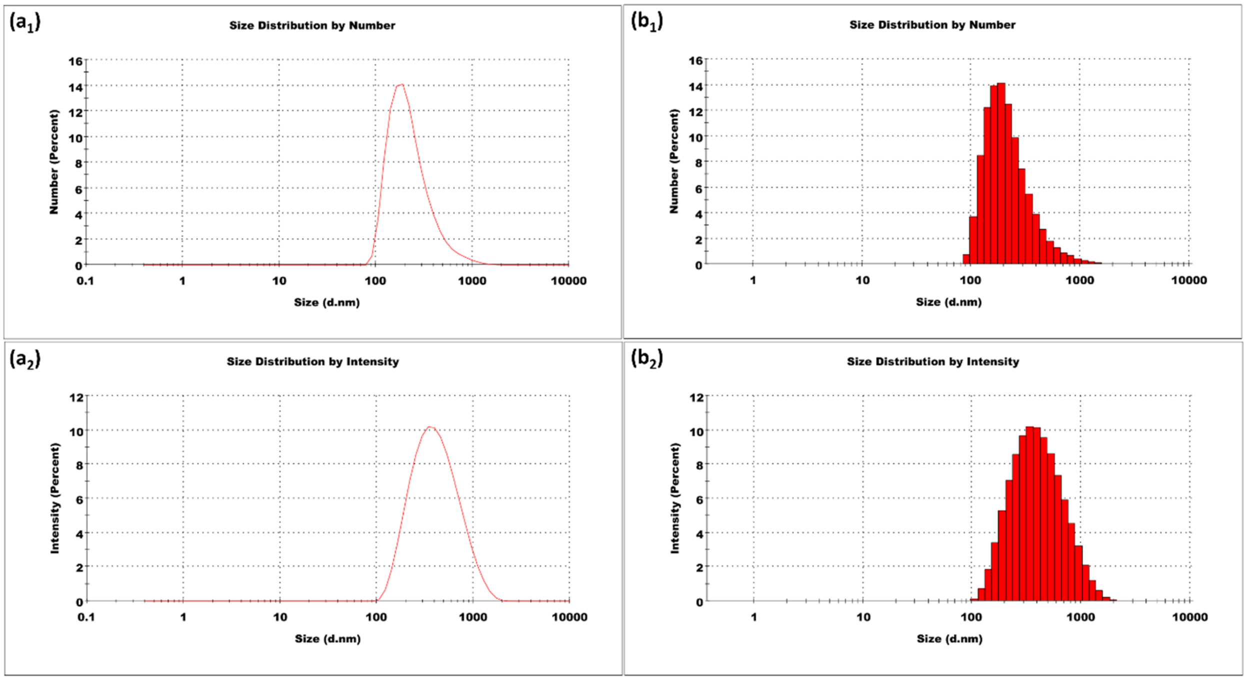

Figure S17. CSNPs synthesized at 80 °C with a CS: TPP ratio of 2:1. The final suspension was analyzed directly. (a1) Raw DLS curve showing Size distribution by number percentage; (a2) Raw DLS curve showing Size distribution by

intensity percentage. (b1) Histogram showing Size distribution by number percentage; (b2) Histogram showing Size distribution by intensity percentage.

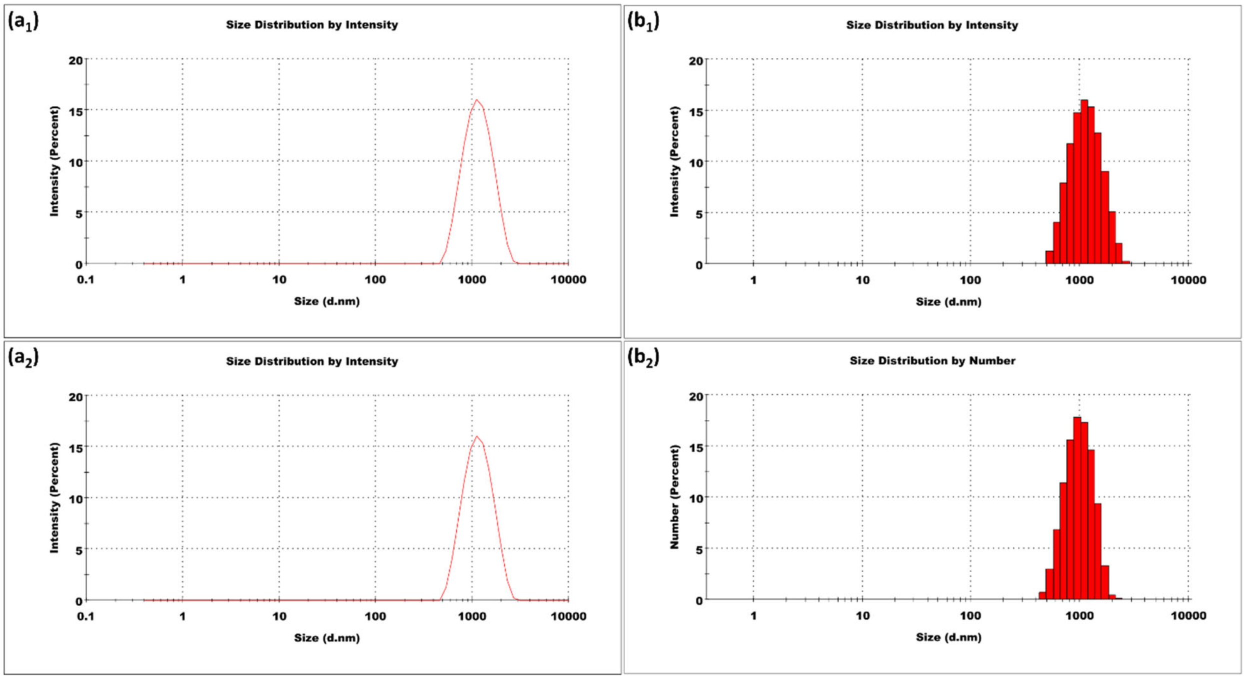

Figure S18. CSNPs synthesized at 80 °C with a CS: TPP ratio of 1.43:1. The final suspension was analyzed directly. (a1) Raw DLS curve showing Size distribution by number percentage; (a2) Raw DLS curve showing Size distribution by intensity percentage. (b1) Histogram showing Size distribution by number percentage; (b2) Histogram showing Size distribution by intensity percentage.

Table S7. CSNPs (5:1) at variable CS: TPP ratio: mean size, mean PDI, SD, and SE.

T (°C)	CS $\frac{mg}{mL}$	TPP $\frac{mg}{mL}$	CS:TPP	Size (nm) xi	Mean (nm) $\bar{x} = \frac{1}{n}\sum_{i=1}^{n} x$	SD $\frac{\sqrt{(xi-\bar{x})}}{n-1}$	SE $\frac{SD}{\sqrt{n}}$	PDI	Mean (nm) $\bar{x} = \frac{1}{n}\sum_{i=1}^{n} x$	SD $\frac{\sqrt{(xi-\bar{x})^2}}{n-1}$	SE $\frac{SD}{\sqrt{n}}$
80	2	0.4	5:1	313	310.3	±3.78	±2.18	0.412	0.429	±0.0147	±0.0085
				312				0.439			
				306				0.436			
80	2	0.6	3.34:1	244	245	±1.73	±1	0.290	0.279	±0.0128	±0.0074
				247				0.265			
				244				0.283			
80	2	0.8	2.5:1	187	188	±1	±0.57	0.189	0.184	±0.0072	±0.0041
				189				0.176			
				188				0.188			
80	2	0.1	2:1	324	324.6	±1.154	±0.66	0.247	0.258	±0.0125	±0.0072
				326				0.257			
				324				0.272			
80	2	0.14	1.43:1	951	937.3	±14.57	±8.41	0.207	0.145	±0.0534	±0.0308
				939				0.112			
				922				0.117			

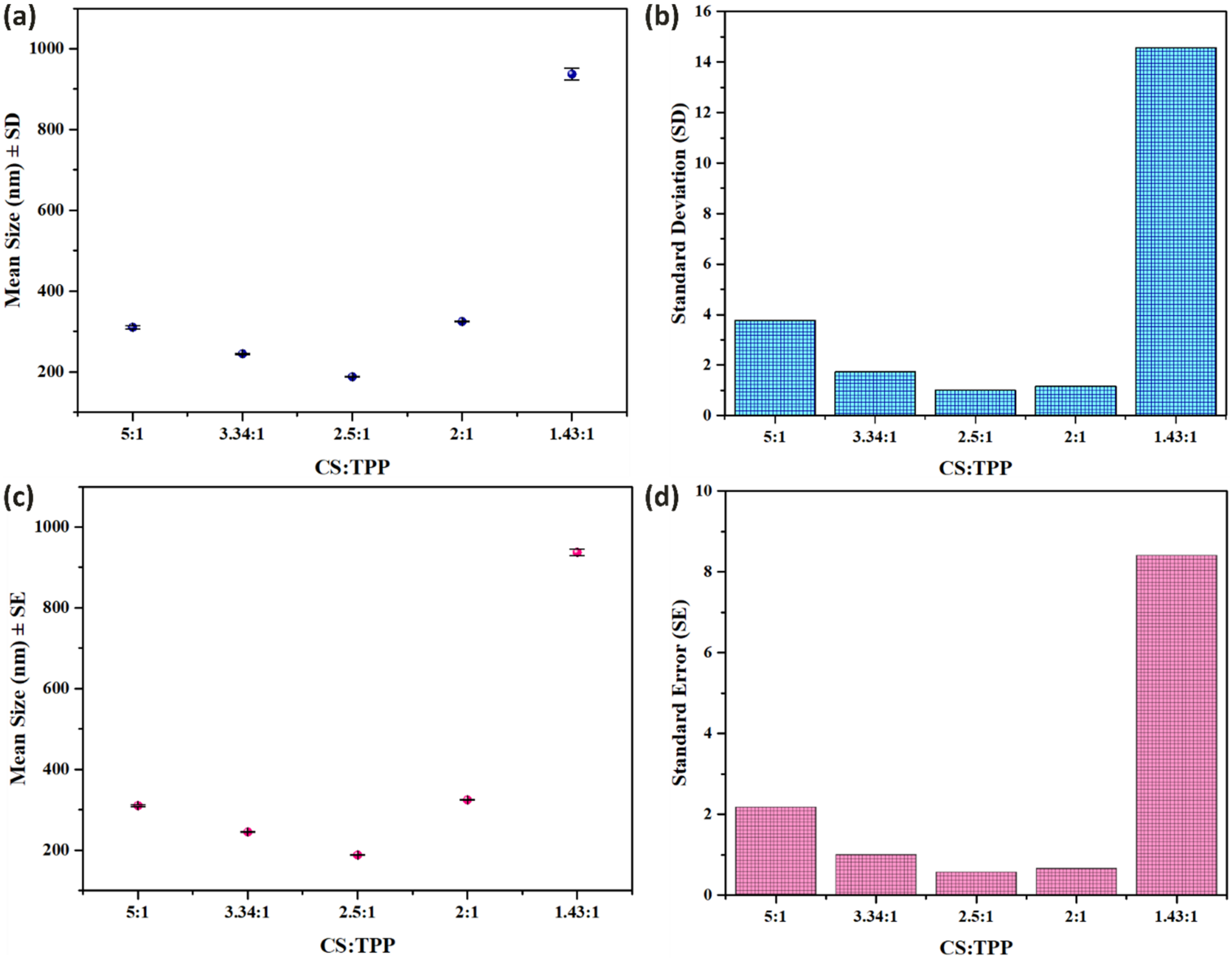

Figure S19. CSNPs with a CS: TPP ratio of 5:1, 3.34:1, 2.5:1, 2:1, and 1.43:1, synthesized at 80 °C using a counter flow microfluidic device, with replication performed three times. (a) Mean size ± SD; (b) SD; (c) Mean size ± SE; (d) SE.

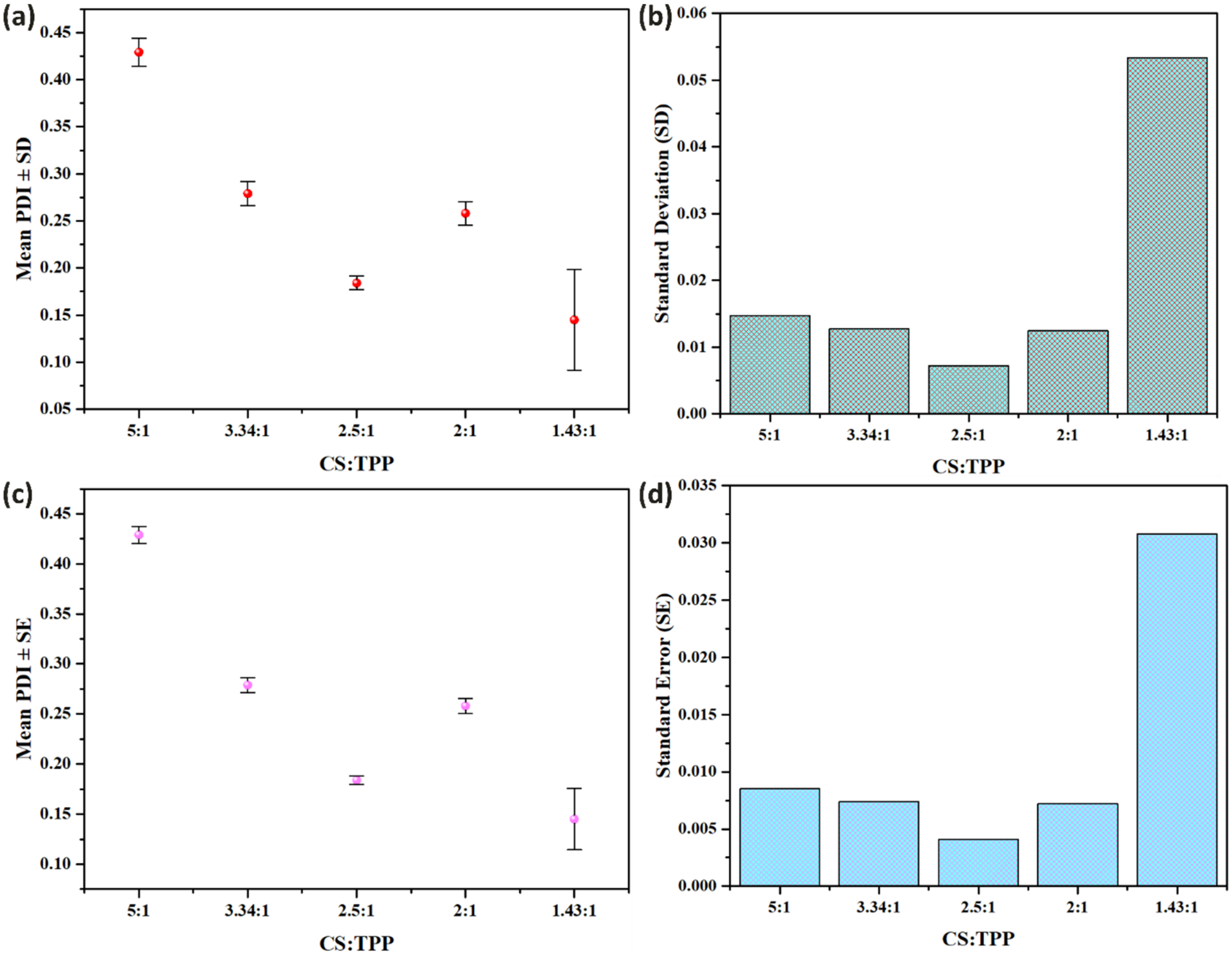

Figure S20. CSNPs with a CS: TPP ratio of 5:1, 3.34:1, 2.5:1, 2:1, and 1.43:1, synthesized at 80 °C using a counter flow microfluidic device, with replication performed three times. (a) Mean PDI ± SD; (b) SD; (c) Mean PDI ± SE; (d) SE.

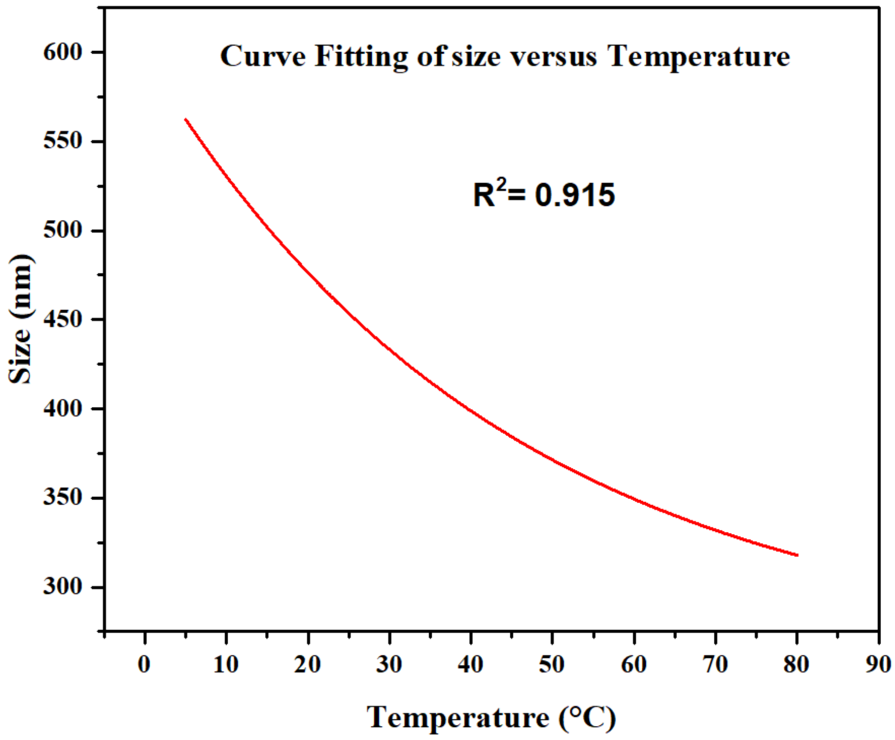

Figure S21. Representation of Size vs. Temperature with exponential fit

References

[1] T.T. Dongsar, T.S. Dongsar, N. Gupta, W.H. Almalki, A. Sahebkar, P. Kesharwani, Emerging potential of 5-Fluorouracil-loaded chitosan nanoparticles in cancer therapy, Journal of Drug Delivery Science and Technology, 82 (2023) 104371.

[2] M.A. Khan, M. Zafaryab, S.H. Mehdi, I. Ahmad, M.M. Rizvi, Characterization and anti-proliferative activity of curcumin loaded chitosan nanoparticles in cervical cancer, Int J Biol Macromol, 93 (2016) 242-253.

[3] T. Nam, S. Park, S.-Y. Lee, K. Park, K. Choi, I.C. Song, M.H. Han, J.J. Leary, S.A. Yuk, I.C. Kwon, Tumor targeting chitosan nanoparticles for dual-modality optical/MR cancer imaging, Bioconjugate chemistry, 21 (2010) 578-582.

[4] G. Chen, Y. Zhao, Y. Xu, C. Zhu, T. Liu, K. Wang, Chitosan nanoparticles for oral photothermally enhanced photodynamic therapy of colon cancer, International Journal of Pharmaceutics, 589 (2020) 119763.

[5] M.A. Khan, M. Zafaryab, S.H. Mehdi, J. Quadri, M.M.A. Rizvi, Characterization and carboplatin loaded chitosan nanoparticles for the chemotherapy against breast cancer in vitro studies, International journal of biological macromolecules, 97 (2017) 115-122.

[6] K. Prabahar, U. Udhumansha, M. Qushawy, Optimization of thiolated chitosan nanoparticles for the enhancement of in vivo hypoglycemic efficacy of sitagliptin in streptozotocin-induced diabetic rats, Pharmaceutics, 12 (2020) 300.

[7] Z. Ma, T.M. Lim, L.-Y. Lim, Pharmacological activity of peroral chitosan–insulin nanoparticles in diabetic rats, International journal of pharmaceutics, 293 (2005) 271-280.

[8] A. Abdel-Moneim, A. El-Shahawy, A.I. Yousef, S.M. Abd El-Twab, Z.E. Elden, M. Taha, Novel polydatin-loaded chitosan nanoparticles for safe and efficient type 2 diabetes therapy: In silico, in vitro and in vivo approaches, International journal of biological macromolecules, 154 (2020) 1496-1504.

[9] F. Jabbari, M.R. Farani, A. Abednejad, B. Akbari, E. Mostafavi, I. Zare, Chitosan nanoparticles in tissue engineering and regenerative medicine, Fundamentals and Biomedical Applications of Chitosan Nanoparticles, Elsevier2025, pp. 497-526.

[10] R. Raftery, F.J. O'Brien, S.-A. Cryan, Chitosan for gene delivery and orthopedic tissue engineering applications, Molecules, 18 (2013) 5611-5647.

[11] A.-M. Safer, S. Leporatti, Chitosan nanoparticles for antiviral drug delivery: A novel route for COVID-19 treatment, International Journal of Nanomedicine, (2021) 8141-8158.
[12] K. Tabynov, M. Solomadin, N. Turebekov, M. Babayeva, G. Fomin, G. Yadagiri, S. Renu, T. Yerubayev, N. Petrovsky, G.J. Renukaradhya, An intranasal vaccine comprising SARS-CoV-2 spike receptor-binding domain protein entrapped in mannose-conjugated chitosan nanoparticle provides protection in hamsters, Scientific Reports, 13 (2023) 12115.
[13] A. Jamali, F. Mottaghitalab, A. Abdoli, M. Dinarvand, A. Esmailie, M.T. Kheiri, F. Atyabi, Inhibiting influenza virus replication and inducing protection against lethal influenza virus challenge through chitosan nanoparticles loaded by siRNA, Drug Delivery and Translational Research, 8 (2018) 12-20.
[14] C. Sawaengsak, Y. Mori, K. Yamanishi, A. Mitrevej, N. Sinchaipanid, Chitosan nanoparticle encapsulated hemagglutinin-split influenza virus mucosal vaccine, AAPS PharmSciTech, 15 (2014) 317-325.
[15] K. Zhao, X. Shi, Y. Zhao, H. Wei, Q. Sun, T. Huang, X. Zhang, Y. Wang, Preparation and immunological effectiveness of a swine influenza DNA vaccine encapsulated in chitosan nanoparticles, Vaccine, 29 (2011) 8549-8556.
[16] S. Dhakal, S. Renu, S. Ghimire, Y. Shaan Lakshmanappa, B.T. Hogshead, N. Feliciano-Ruiz, F. Lu, H. HogenEsch, S. Krakowka, C.W. Lee, Mucosal immunity and protective efficacy of intranasal inactivated influenza vaccine is improved by chitosan nanoparticle delivery in pigs, Frontiers in immunology, 9 (2018) 934.
[17] K. Zhao, G. Chen, X.-m. Shi, T.-t. Gao, W. Li, Y. Zhao, F.-q. Zhang, J. Wu, X. Cui, Y.-F. Wang, Preparation and efficacy of a live newcastle disease virus vaccine encapsulated in chitosan nanoparticles, PloS one, 7 (2012) e53314.
[18] J.A.J. Nesalin, A.A. Smith, Preparation and evaluation of stavudine loaded chitosan nanoparticles, Journal of Pharmacy Research, 6 (2013) 268-274.
[19] T. Hunsawong, P. Sunintaboon, S. Warit, B. Thaisomboonsuk, R.G. Jarman, I.-K. Yoon, S. Ubol, S. Fernandez, Immunogenic properties of a BCG adjuvanted chitosan nanoparticle-based dengue vaccine in human dendritic cells, PLoS neglected tropical diseases, 9 (2015) e0003958.
[20] C. Prego, P. Paolicelli, B. Díaz, S. Vicente, A. Sánchez, Á. González-Fernández, M.J. Alonso, Chitosan-based nanoparticles for improving immunization against hepatitis B infection, Vaccine, 28 (2010) 2607-2614.

[21] L. Wang, Y. Zhao, Y. Wang, F. Zhang, Y. Wei, N. Li, Y. Xu, Preparation, stability, and antibacterial activity of carboxymethylated Anemarrhena asphodeloides polysaccharide-chitosan nanoparticles loaded curcumin, International Journal of Biological Macromolecules, 264 (2024) 130787.
[22] Y. Liu, J. Chen, H. Li, Y. Wang, Nanocomplexes film composed of gallic acid loaded ovalbumin/chitosan nanoparticles and pectin with excellent antibacterial activity: Preparation, characterization and application in coating preservation of salmon fillets, International Journal of Biological Macromolecules, 259 (2024) 128934.
[23] W. Xu, D.J. McClements, Z. Zhang, R. Zhang, C. Qiu, J. Zhao, Z. Jin, L. Chen, Effect of tannic acid modification on antioxidant activity, antibacterial activity, environmental stability and release characteristics of quercetin loaded zein-carboxymethyl chitosan nanoparticles, International Journal of Biological Macromolecules, 280 (2024) 135853.
[24] Z. Wang, M. Zhang, S. Liang, Y. Li, Enhanced antioxidant and antibacterial activities of chitosan/zein nanoparticle Pickering emulsion-incorporated chitosan coatings in the presence of cinnamaldehyde and tea polyphenol, International Journal of Biological Macromolecules, 266 (2024) 131181.
[25] J. Diaz-Ramirez, S. Basasoro, S. Torresi, A. Eceiza, A. Retegi, N. Gabilondo, Bacterial cellulose/thiolated chitosan nanoparticles hybrid antimicrobial dressing for curcumin delivery, International Journal of Biological Macromolecules, (2024) 138836.
[26] M. Derakhshan-Sefidi, B. Bakhshi, A. Rasekhi, Thiolated chitosan nanoparticles encapsulated nisin and selenium: antimicrobial/antibiofilm/anti-attachment/immunomodulatory multi-functional agent, BMC microbiology, 24 (2024) 257.
[27] S. Revathi, N. Dey, S. Thangaleela, S. Vinayagam, L. Gnanasekaran, T. Sundaram, A. Malik, A.A. Khan, A. Roy, A. Kumar, Nanocarrier optimization: Encapsulating Hydrastis canadensis in chitosan nanoparticles for enhanced antibacterial and dye degradation performance, International Journal of Biological Macromolecules, (2024) 133316.
[28] X. Jiang, Y. Yu, S. Ma, L. Li, M. Yu, M. Han, Z. Yuan, J. Zhang, Chitosan nanoparticles loaded with Eucommia ulmoides seed essential oil: Preparation, characterization, antioxidant and antibacterial properties, International Journal of Biological Macromolecules, 257 (2024) 128820.
[29] R. Nayak, J. Halder, T.K. Rajwar, D. Pradhan, P. Dash, C. Das, V.K. Rai, B. Kar, G. Ghosh, G. Rath, Design and evaluation of antibacterials crosslinked chitosan nanoparticle as a novel

carrier for the delivery of metronidazole to treat bacterial vaginosis, Microbial Pathogenesis, 186 (2024) 106494.
[30] F. Gláucia-Silva, J.V.P. Torres, M. Torres-Rêgo, A. Daniele-Silva, A.A. Furtado, S.d.S. Ferreira, G.M. Chaves, F.H. Xavier-Júnior, K.S. Rocha Soares, A.A.d. Silva-Júnior, Tityus stigmurus-Venom-Loaded Cross-Linked Chitosan Nanoparticles Improve Antimicrobial Activity, International Journal of Molecular Sciences, 25 (2024) 9893.
[31] R. Li, J. He, H. Xie, W. Wang, S.K. Bose, Y. Sun, J. Hu, H. Yin, Effects of chitosan nanoparticles on seed germination and seedling growth of wheat (Triticum aestivum L.), International journal of biological macromolecules, 126 (2019) 91-100.
[32] R.C. Choudhary, A. Joshi, S. Kumari, R. Kumaraswamy, V. Saharan, Preparation of Cu-chitosan nanoparticle and its effect on growth and enzyme activity during seed germination in maize, Journal of Pharmacognosy and Phytochemistry, 6 (2017) 669-673.
[33] V. Saharan, R. Kumaraswamy, R.C. Choudhary, S. Kumari, A. Pal, R. Raliya, P. Biswas, Cu-chitosan nanoparticle mediated sustainable approach to enhance seedling growth in maize by mobilizing reserved food, Journal of agricultural and food chemistry, 64 (2016) 6148-6155.
[34] M. Mondéjar-López, A. Rubio-Moraga, A.J. López-Jimenez, J.C.G. Martínez, O. Ahrazem, L. Gómez-Gómez, E. Niza, Chitosan nanoparticles loaded with garlic essential oil: A new alternative to tebuconazole as seed dressing agent, Carbohydrate polymers, 277 (2022) 118815.
[35] Z. Shirkhani, A. Chehregani Rad, F. Mohsenzadeh, Improving Cd‐phytoremediation ability of Datura stramonium L. by Chitosan and Chitosan nanoparticles, Biologia, 76 (2021) 2161-2171.
[36] P.L. Kashyap, X. Xiang, P. Heiden, Chitosan nanoparticle based delivery systems for sustainable agriculture, International journal of biological macromolecules, 77 (2015) 36-51.
[37] M.E.A. Ali, M.M.S. Aboelfadl, A.M. Selim, H.F. Khalil, G.M. Elkady, Chitosan nanoparticles extracted from shrimp shells, application for removal of Fe (II) and Mn (II) from aqueous phases, Separation Science and Technology, 53 (2018) 2870-2881.
[38] U. Haripriyan, K. Gopinath, J. Arun, Chitosan based nano adsorbents and its types for heavy metal removal: A mini review, Materials Letters, 312 (2022) 131670.
[39] Z. Hu, J. Zhang, W. Chan, Y. Szeto, The sorption of acid dye onto chitosan nanoparticles, Polymer, 47 (2006) 5838-5842.

[40] I.M. Lipatova, L.I. Makarova, A.A. Yusova, Adsorption removal of anionic dyes from aqueous solutions by chitosan nanoparticles deposited on the fibrous carrier, Chemosphere, 212 (2018) 1155-1162.

[41] H. Momenzadeh, A.R. Tehrani-Bagha, A. Khosravi, K. Gharanjig, K. Holmberg, Reactive dye removal from wastewater using a chitosan nanodispersion, Desalination, 271 (2011) 225-230.
